# Trem2 acts as a non‐classical receptor of interleukin‐4 to promote diabetic wound healing

**DOI:** 10.1002/ctm2.70026

**Published:** 2024-09-30

**Authors:** Xinlin Zhu, Chao Zhang, Weiwei Jiang, Zhaoxiang Zeng, Keming Zhang, Mingwei Du, Juan Chen, Qian Wu, Wanqing Liao, Youming Chen, Wenjie Fang, Weihua Pan

**Affiliations:** ^1^ Department of Dermatology Shanghai Key Laboratory of Medical Mycology; The Center for Basic Research and Innovation of Medicine and Pharmacy (MOE) Shanghai Changzheng Hospital Naval Medical University Shanghai China; ^2^ Department of Vascular Surgery Shanghai General Hospital, School of Medicine Shanghai Jiaotong University Shanghai China; ^3^ Department of Laboratory Medicine Tongren Hospital Shanghai Jiao Tong University School of Medicine Shanghai China; ^4^ Department of Infectious Diseases and Immunology Shanghai Public Health Clinical Center Fudan University Shanghai China

**Keywords:** diabetic wound healing, IL‐4, macrophage, MAPK, Trem2

## Abstract

**Background:**

The immunoglobulin superfamily protein Trem2 (triggering receptor expressed on myeloid cells 2) is primarily expressed on myeloid cells where it functions to regulate macrophage‐related immune response induction. While macrophages are essential mediators of diabetic wound healing, the specific regulatory role that Trem2 plays in this setting remains to be established.

**Objective:**

This study was developed to explore the potential importance of Trem2 signalling in diabetic wound healing and to clarify the underlying mechanisms through which it functions.

**Methods and results:**

Following wound induction, diabetic model mice exhibited pronounced upregulation of Trem2 expression, which was primarily evident in macrophages. No cutaneous defects were evident in mice bearing a macrophage‐specific knockout of Trem2 (T2‐cKO), but they induced more pronounced inflammatory responses and failed to effectively repair cutaneous wounds, with lower levels of neovascularization, slower rates of wound closure, decreased collagen deposition following wounding. Mechanistically, we showed that interleukin (IL)‐4 binds directly to Trem2, inactivating MAPK/AP‐1 signalling to suppress the expression of inflammatory and chemoattractant factors. Co‐culture of fibroblasts and macrophages showed that macrophages from T2‐cKO mice suppressed the in vitro activation and proliferation of dermal fibroblasts through upregulation of leukaemia inhibitory factor (Lif). Injecting soluble Trem2 in vivo was also sufficient to significantly curtail inflammatory responses and to promote diabetic wound healing.

**Conclusions:**

These analyses offer novel insight into the role of IL‐4/Trem2 signalling as a mediator of myeloid cell‐fibroblast crosstalk that may represent a viable therapeutic target for efforts to enhance diabetic wound healing.

## Graphical abstract

This study elucidates the pivotal role of TREM2 in diabetic wound healing. This study identified the key regulatory gene involved in diabetic wound healing as TREM2. This study has found that IL‐4 functions as an upstream ligand, activating TREM2. This study provides Evidence of the regulatory effects of macrophages by IL‐4/TREM2/AP‐1/LIF pathway on fibroblasts. This study validated the therapeutic potential using the sTREM2.

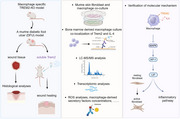

## 1 INTRODUCTION

Diabetes mellitus (DM) is a metabolic disorder characterized by hyperglycaemia due to abnormal insulin production or functionality.^1^, ^2^ Patients with DM experience pronounced reductions in their quality of life and often exhibit poorer survival outcomes as compared to the general population. As DM can impair wound healing, chronic wounds are a common finding in DM patients.^1^ Standard wound care and infection control methods in these cases generally consist of a combination of debridement, pressure relief through off‐loading and the moistening of the wound bed. This approach, however, generally fails to achieve the desired efficacy in individuals with DM, underscoring a need for more effective therapeutic interventions.^3^, ^4^

The process of diabetic wound healing is a multi‐stage process that entails interplay between inflammatory, re‐epithelialization, vascularization and regenerative processes.^1^, ^5^ Local macrophages within the tissue microenvironment are particularly important regulators of such wound healing,^6^ functioning to eliminate potentially pathogenic microbes, terminate inflammatory responses, and promote the induction and progression of tissue regeneration and remodelling.^6^ Tissue macrophages generally undergo a transition from a pro‐inflammatory phenotype to an anti‐inflammatory phenotype over the course of wound healing.^7^ The resultant anti‐inflammatory macrophages localize proximal to developing blood vessels where they can secrete Vascular endothelial growth factor (VEGF), Transforming growth factor β (TGF‐β) or other cytokines that can promote angiogenic activity and wound closure.^8^ A growing body of evidence suggests that macrophages can also engage in crosstalk with fibroblasts through inflammatory cytokine production, with these regulatory interactions being important for wound repair and associated fibrotic activity.^9^ In the final phases of tissue remodelling, macrophages can reduce or modulate extracellular matrix deposition by secreting matrix metalloproteinases and can also induce the senescence of dermal fibroblasts and other local stem cell populations such that the wounded tissue is restored to its mature pre‐wounding status.^10^

The transmembrane receptor protein Trem2 (triggering receptor expressed on myeloid cells 2) is primarily expressed by macrophage and microglial cell populations.^11^, ^12^ Prior research has clearly established a link between Trem2 and the risk of Alzheimer's disease (AD). And it comes out Trem2 regulates lipid metabolism both in the central nervous system (CNS) and the periphery. Meanwhile, these studies have indicated the inseparable relationship between Trem2 and cell metabolism, providing crucial foundational information for our research.^11^, ^12^ Within the CNS, Trem2 serves as a receptor that can detect a range of ligand molecules including lipids, lipoproteins and oligomeric amyloid‐β, all of which become dysregulated in the context of AD development.^13^ After binding to its cognate ligands, Trem2 heterodimerizes with the adaptor proteins DNAX activation protein 10 (DAP10) and DAP12, after which downstream signalling activity is induced via phosphatidylinositol‐specific phospholipase Cγ2, cleaving the membrane phospholipid phosphatidylinositol‐4,5‐bisphosphate (PIP2) to generate diacylglycerol (DAG) and inositol‐1,4,5‐trisphosphate (IP_3_).^11^, ^12^ In the CNS, Trem2 is able to bind the key lipid transporter apolipoprotein E.^14^ Trem2‐related disruptions of lipid metabolic activity generally drive otherwise normal microglia to transition into disease‐associated microglia.^11^ Trem2 can also play a role in shaping lipid metabolic activity in the periphery, particularly in diseases associated with abnormal lipid metabolic activity resulting in the excessive biogenesis, accumulation or lipolysis of these lipid molecules.^11^, ^15^ Jaitin et al identify conserved Trem^+^ lipid‐associated macrophages, wherein Trem2 deletion in mice inhibits macrophage association with lipids and results in system hypercholesterolemia, fat accumulation and glucose intolerance.^16^ Wang et al. indicated Trem2 expression in macrophages is required for efferocytosis of lipid‐laden apoptotic hepatocytes, thus regulating liver homeostasis and important to fight back against conditions such as non‐alcoholic steatohepatitis.^17^ Moreover, previous studies have reported that Trem2 plays a key role in the repair of the colonic mucosa, and the expression of interleukin (IL)‐4 changes after Trem2 knockout.^18^ Peripheral Trem2‐mediated signalling pathways and the importance of these signalling mechanisms in pathogenic states in the periphery, however, are not well understood and have yet to be characterized in the context of diabetic wound healing.

This study was developed to explore the effects of the loss of macrophage Trem2 expression on the process of skin wound healing in a diabetic model system and to clarify the underlying mechanism. These experiments were conducted using diabetic mice harbouring a macrophage‐specific deletion of Trem2 (T2‐cKO), providing a means of directly testing the importance of Trem2 in this myeloid compartment over the course of diabetic skin wound healing. Importantly, the detailed mechanistic studies in macrophages showed that IL‐4 binds directly to Trem2 to block both MAPK/AP‐1 signalling and the production of anti‐inflammatory cytokines. These findings identify that the IL‐4/Trem2 interaction plays a critical role in diabetic wound healing.

## 2 MATERIALS AND METHODS

### 2.1 Animal model

The Animal Ethics Committee of the Shanghai Changzheng Hospital approved all animal studies described herein. A C57BL/6J background Trem2^flox/flox^ and Lyz2‐Cre mice were obtained from the Shanghai Model Organisms Center and crossed for 2−3 generations to establish mice bearing the conditional macrophage‐specific deletion of Trem2 (Trem2^flox/flox^Lyz2‐Cre or T2‐cKO) and wild‐type (T2‐flox) littermate controls. Genotyping of Trem2‐cKO and Lyz2‐cre mice is shown in Supplementary Material S1.

To establish a murine diabetic foot ulcer (DFU) model system, diabetic mice (male, body weight: 20−24 g, 8−12 weeks old) were established by single intraperitoneal injection of 155 mg/kg streptozotocin in 100 µL normal saline after a 12‐h fasting, with those animals exhibiting blood glucose levels ≥ 16.7 mM on 10 consecutive days being considered diabetic model mice. These mice were anesthetized and their abdominal area was shaved, after which a sterile punch was used to generate a wound .8 cm in diameter. Immediately after wound creation, soluble Trem2 (sTrem2) was applied both to the wound bed and the surrounding area. The therapeutic effects of treating these wounds with sTrem2 were examined by injecting 12 µg of murine sTrem2 (mouse Trem2aa 12−171 His_N‐term; Lifespan BioSciences), Phosphate buffer saline (PBS) or gel into two points on the skin surrounding the wound site (10 µL/injection). The wound edges were delineated on transparent film to establish the wound area, with wound healing being evaluated via light microscopy on different days. Briefly, photo‐documentation of wound closure in all groups was carried out. Using digital images and Image J toolkit, we monitored the wound and the area around the wound every day. From these measurements, we calculated the percent total wound closure (%TWC) as: [(WA_0_ − WA_T_)/ WA_0_] ×100% where WA_0_ = wound area at day 0 and WA_T_ = wound area at time point T.

### 2.2 Histological and immunostaining analyses

The samples of wound tissue were harvested from these DFU model mice, fixed with 10% paraformaldehyde, paraffin‐embedded and cut into 5−15 µm‐thick sections. Haematoxylin and eosin (H&E) staining was then performed by deparaffinizing and rehydrating these samples, staining them with H&E solutions, covering them with Permount solution, and allowing them to dry overnight. Masson's trichrome staining was performed by deparaffinizing and rehydrating these samples, after which they were stained using Weigert's iron haematoxylin, Biebrich scarlet‐acid fuchsin and aniline blue solutions. Sections were dehydrated following rinsing and were mounted using a resin‐based medium.

The tissues underwent fixation in neutral formalin for 48 h, followed by paraffin embedding and slicing into 5‐micrometer‐thick sections. Dewaxing and hydration procedures were employed to remove paraffin residues from the tissue slices. Utilizing Sodium Citrate Antigen Retrieval Solution, antigen retrieval was carried out (C02‐02002, Bioss Antibodies). By soaking the slides in 3% hydrogen peroxide (10011218, G‐clone) for 10 min, the endogenous peroxidase activity was inhibited. The sections were then blocked for 30 min at 37°C in a blocking solution containing 5% BSA (A8020, Solarbio) and .1% Triton X‐100 (Boster Biological Technology Co., Ltd.). Primary antibodies were incubated on the section for an overnight period at 4°C. Following that, each segment was incubated with secondary antibodies at 37°C for 30 min. Slides were washed in PBS and then developed using a 3,3ʹ‐diaminobenzidine (DAB) substrate kit, then slides were covered in haematoxylin for 3 min and counterstained in PBS for 5∼10 min. The slides were dehydrated and coverslipped using an aqueous mounting medium. Slides were dried in a fume hood overnight. Pictures were taken using a digital section scanner (Pannoramic MIDI II, 3DHISTECH). The quantitative assessments were conducted blindly using the Image J toolkit by multiple authors to ensure accuracy and reproducibility.

For the immunofluorescence (IF) staining protocol, the tissue was first blocked with 5% BSA to minimize non‐specific binding. Following this, the sections were stained with a primary antibody and incubated overnight at 4°C to ensure thorough antigen recognition. Subsequently, a secondary antibody was applied to the sections, allowing for fluorescent signal amplification. 4′,6‐diamidino‐2‐phenylindole (DAPI) was employed as a nuclear counterstain, diluted at a ratio of 1:1000, and incubated for 5 min to enhance nuclear visualization. The immunology‐positive cells were carefully examined and captured using a fluorescence microscope (Nikon) at a magnification of 200×. Detailed information on the primary antibodies utilized for these analyses is provided in Supplementary Material S2A.

### 2.3 Bone‐marrow‐derived macrophage culture

Male mice aged 6−12 weeks were euthanized and bone marrow cells were collected, passed through a 100‐µm cell strainer, and counted prior to the suspension of ∼1×10^7^ live cells in bone marrow culture media (IMDM + 10% foetal bovine serum + 1% penicillin‐streptomycin + 10 ng/mL macrophage colony‐stimulating factor [M‐CSF]). These cells were then transferred into 12‐well plates and incubated in a 5% CO_2_ tissue culture incubator at 37°C. On day 3, media was exchanged for fresh culture media. Anti‐inflammatory phenotype was induced by stimulating these bone‐marrow‐derived macrophages (BMDMs) with IL‐4 (40 ng/mL) for 48 h beginning on day 7.

### 2.4 Analyses of anti‐inflammatory macrophage phenotype and co‐localization of Trem2 and IL‐4 in macrophages

IL‐4‐stimulated BMDMs using .25% EDTA‐trypsin and transferring 1−1.5×10^4^ cells onto individual coverslips placed in 12‐well plates, with a final volume of 500 µL of media per well. After culturing for 36 h, media was removed, cells were rinsed three times with PBS (500 µL/wash), fixed using 4% paraformaldehyde, permeabilized using .1% Triton X‐100 in PBS and blocked using 1% BSA. Cells were then stained with appropriate primary antibodies, fluorescently conjugated secondary antibodies and DAPI. Anti‐inflammatory phenotype was assessed by staining for F4/80, CD206 and Arg1. The co‐localization of Trem2 (green) and IL‐4 (red) was evaluated in BMDMs.

### 2.5 Murine skin fibroblast harvesting and culture

Dermal tissue samples were harvested from postnatal mice and minced with a sterile scalpel prior to digestion for a minimum of 1 h in .25% collagenase at 37°C prior to passage through a 70‐µm filter. The resultant single‐cell suspension was then transferred into a 75 cm^2^ tissue culture flask containing fibroblast culture media (DMEM + 20% FBS + 1% penicillin‐streptomycin) and incubated in a tissue culture incubator (5% CO_2_, 37°C). On day 3, media was exchanged for fresh media while leaving adherent fibroblasts intact. Cells were subsequently expanded, passaged or cryopreserved as appropriate.

Wound healing assays were performed by plating fibroblasts in 12‐well plates (2×10^6^ cells/mL). When these cells were 70−80% confluent, a straight scratch wound was generated in the monolayer with a 1 mm pipette tip, after which the surface was rinsed to remove non‐adherent cells, and fresh medium was added to each well. Wound closure was then imaged every 12 h with a phase‐contrast microscope until the wound had fully closed.

Transwell assays were performed by harvesting fibroblasts with .25% EDTA‐trypsin, rinsing them three times with PBS and resuspending them at 1×10^6^ cells/mL in serum‐free DMEM. A total of 100 µL of these cells (1×10^5^ cells) were then transferred into the upper portion of a transwell insert (8‐µm pore size; 24‐well transwell plate), while the lower chamber was filled with complete culture medium containing 10% FBS. Plates were subsequently incubated for 48 h, after which inserts were removed and cells on the lower membrane surface were fixed for 10 min using 4% paraformaldehyde, stained for 20 min with 1% crystal violet, rinsed repeatedly with PBS, and cells on the upper membrane surface were removed with a cotton swab. When membranes had fully dried, a microscope was used to count cells on the lower membrane surface.

Alpha‐smooth muscle actin (α‐SMA) immunostaining was performed by seeding fibroblasts in 12‐well plates containing coverslips. When these cells were 60−70% confluent, coverslips were rinsed three times using chilled PBS, after which cells were fixed with 4% paraformaldehyde and stained with primary anti‐αSMA and appropriate fluorescently conjugated secondary antibodies. When assessing the proliferation of these cells, culture media was supplemented with EdU Cell Proliferation Kit (Sangon Biotech, E607204) for 2 h prior to fixation.

### 2.6 Macrophage and fibroblast co‐culture assays

Supernatants were harvested from pro‐inflammatory or anti‐inflammatory macrophages and passed through a .45‐µm membrane filter (Millipore‐Sigma), after which primary dermal fibroblasts were cultured for 24–48 h in this conditioned media.

### 2.7 Reactive oxygen species analyses

Reactive oxygen species (ROS) levels were analysed in cells via dihydroethidium (DHE) staining. Briefly, on the day before the experiment, cells were plated in a live cell imaging culture dish. The following day, culture media was abandoned, cells were rinsed with PBS, stained for 1 h with 1 µmol/L DHE, rinsed twice with PBS and imaged via fluorescent microscopy. Skin tissue ROS levels were also analysed by using DHE as a fluorescent probe, with harvested samples being stained for 45 min with DHE at 37°C in a dark humidified chamber.

### 2.8 Lentiviral transduction

PLKO.1‐puro plasmids harbouring shRNA sequences specific for Junb and Fos1 were constructed and co‐transfected into HEK‐293T cells along with the psPAX2 and pMD2.G vectors using the FuGENE 6 transfection reagent. At 48 h post‐transfection, media was collected from these cells, passed through a .45‐µm membrane filter, and lentiviral particles were collected via ultracentrifugation. Lentiviral titres were determined using a limiting dilution approach, and particles were diluted at a final concentration of 1×10^7^ TU/mL. Macrophages stably expressing Fos1 or Junb‐specific shRNA constructs were established by infecting healthy M0 BMDMs with these lentiviruses for 48 h, after which puromycin was added to select for successfully transduced cells. The in vivo knockdown of these genes was achieved by injecting these lentiviral particles around the wound sites in DFU model mice.

### 2.9 ELISAs

Sandwich ELISA kits from RayBiotech were used to analyse macrophage‐derived IL‐10, VEGF, TGF‐β1 and Platelet derived growth factor (PDGF) concentrations. Briefly, supernatants were harvested from anti‐inflammatory macrophage cultures and transferred into pre‐coated microplates, with detection antibodies and Horseradish Peroxidase (HRP) being added as indicated in the provided instructions prior to colour development, with absorbance values being analysed at 450 nm with a microplate reader. Protein concentrations were established using a standard curve.

### 2.10 Transcriptomic analyses

IL‐4‐stimulated macrophages for 24 h or skin samples collected from DFU model mice on day 3 were lysed with Trizol to extract RNA, after which a Nanodrop spectrophotometer was used to evaluate RNA sample purity and concentration. NEBNext Ultra II RNA Kits were then used for RNA library preparation prior to sequencing with an Illumina HiSeq X10 instrument. The resultant raw data were analysed with DeSeq2 using the GRCh38 reference genome. Differentially expressed genes (DEGs) were further enriched.

### 2.11 Immunoprecipitation and glutathione S‐transferase pull‐down

In the immunoprecipitation assay, lysates were prepared from HEK‐293T cells and RAW 264.7 cells in 1× IP lysis buffer. Immunoprecipitation was then performed by incubation with an anti‐IL‐4 antibody or anti‐Trem2 antibody at 4°C overnight followed by incubation with Protein A/G Plus‐Agarose at 4°C for 2 h with gentle rotation. The beads were washed three times with 2× IP lysis buffer and boiled for 15 min in reducing sodium dodecyl sulphate (SDS) sample buffer. The immunoprecipitated proteins were separated on SDS‐PAGE, with 10% gels used for further western blotting.

For the glutathione S‐transferase (GST) pull‐down assay, GST‐IL‐4 fusion proteins were immobilized on GST beads and incubated with His‐tagged (His‐Trem2, His‐Trem2 [NT‐aa1‐132], His‐Trem2 [NT‐aa133‐227]) binding buffer. The expression of GST fusion proteins was confirmed by SDS‐PAGE with Coomassie Brilliant Blue staining. The interaction of GST‐IL‐4 and His‐Trem2 was confirmed by western blotting.

### 2.12 qPCR

Trizol was used to extract RNA from cell samples, after which RNA purity and concentrations were analysed with a Nanodrop spectrophotometer. A total of 1 µg of RNA per sample was reverse transcribed to produce cDNA, and all qPCR analyses were performed with the SYBR Green qPCR Master Mix kit (Thermo Fisher Scientific). ddCt method was used for qRT–PCR data analysis and the results were normalized to β‐actin expression. The relevant primers used in this section are detailed in Supplementary Material S3.

### 2.13 Western immunoblotting

In total, 1×10^6^ cells per sample were harvested, rinsed with cold PBS and lysed in 100 µL of 1×SDS loading buffer containing 2% SDS to extract protein. These lysates were then boiled for 10 min at 100°C and separated by 10% or 12.5% SDS‐PAGE prior to transfer to PVDF membranes. The information of primary antibodies used to probe blots in this study was specific for proteins and is shown in Supplementary Material S2B.

### 2.14 Flow cytometry analysis

The skin tissues were dissected from the surrounding fascia, weighed, mechanically minced, and then isolated using the whole skin dissociation Kit (mouse, Miltenyi Biotech). To analyse surface markers, cells were suspended in a cell staining buffer (420201, Biolegend). For surface antigen staining, cells were incubated with anti‐mouse CD16/CD32 antibody (Mouse Fc Block, 1:200, 156604, Biolegend) and then incubated with surface proteins antibodies in the dark at 4°C for 30 min. Fixable Viability DyeFixable viability dye (eFluor 450, 1:2000, eBioscience) was used for live/dead discrimination. The antibodies utilized for staining included: anti‐CD45 (1:200, 157607, Biolegend), anti‐CD11b (1:100, 101212, Biolegend), anti‐F4/80 (1:400, 123133, Biolegend), anti‐Ly6G (1:400, 127623, Biolegend), anti‐CD86 (1:400, 105021, Biolegend), anti‐CD206 (1:400, 141719, Biolegend) and anti‐Trem2 (1:200, ab125117, abcam). Flow cytometry data were collected on LSR Fortessa (BD Biosciences) and analysed using FlowJo software (v10.8.1).

### 2.15 LC‐MS/MS analysis

To establish a Trem2 overexpression stable cell line, we employed the Trem2 lentivirus. Macrophage cells were cultured in a medium enriched with a high glucose concentration (25 mM, Gibco, Thermo Fisher Scientific) and then stimulated with Lipopolysaccharides (LPS) at a concentration of 100 ng/mL for 6 h. Subsequently, 1×10^7 cells were collected for immunoprecipitation, utilizing the Immunoprecipitation System (Millipore, 17−500) strictly according to protocol.

After co‐immunoprecipitation, equivalent amounts of proteins were separated on 4−12% SDS‐PAGE gels and visualized with Coomassie blue G250 staining (BioRad). The stained bands were cut into individual fractions, excluding the IgG chains. These fractions were further subdivided and transferred to a 1.5‐mL tube.

The proteins were enzymatically digested with trypsin using a modified version of the filter‐assisted sample preparation protocol.^19^ Briefly, the proteins were loaded into 10 kDa centrifugal filter tubes (Millipore Corporation) and rinsed twice with 200 µL of Urea (UA) buffer (consisting of 8 M urea and .1 M Tris‐HCl, pH 8.5). Next, they were alkylated with 50 mM iodoacetamide in UA buffer for 30 min in the dark at room temperature (RT). After three washes with UA buffer, the proteins were rinsed three times with 200 µL of 50 mM ammonium bicarbonate (ABC). All centrifugation steps were carried out at 12 000 *g* at RT.

The proteins were then digested with trypsin (Promega) in 50 mM ABC and incubated at 37°C for 18 h. Upon completion of digestion, the peptides were concentrated by vacuum centrifugation and desalted using C18 solid‐phase extraction (3 M Empore). The desalted peptides were evaporated again using vacuum centrifugation, and a portion of these dried peptides were prepared for subsequent LC‐MS/MS analysis.

### 2.16 Statistical analysis

The selection of statistical tests was guided by the characteristics of the data, specifically its distribution and homogeneity of variance. In cases where the data exhibited normal distribution and homogeneity of variance, an unpaired two‐tailed Student's *t*‐test was applied for comparing two distinct groups. For scenarios involving multiple groups, a one‐way ANOVA, followed by Dunnett's multiple comparison test, was employed. Additionally, to assess the wound area in relation to both treatment and time, a repeated measures two‐way ANOVA was conducted. The significance levels were represented using asterisks, with *, ** and *** indicating *p*‐values less than .05, .01 and .001, respectively. NS was used to denote the absence of a statistically significant difference. The relevant statistical analysis was done using GraphPad Prism 9 software package.

## 3 RESULTS

### 3.1 Diabetic ulcer patients and mice exhibit upregulated Trem2 expression

**FIGURE 1:**
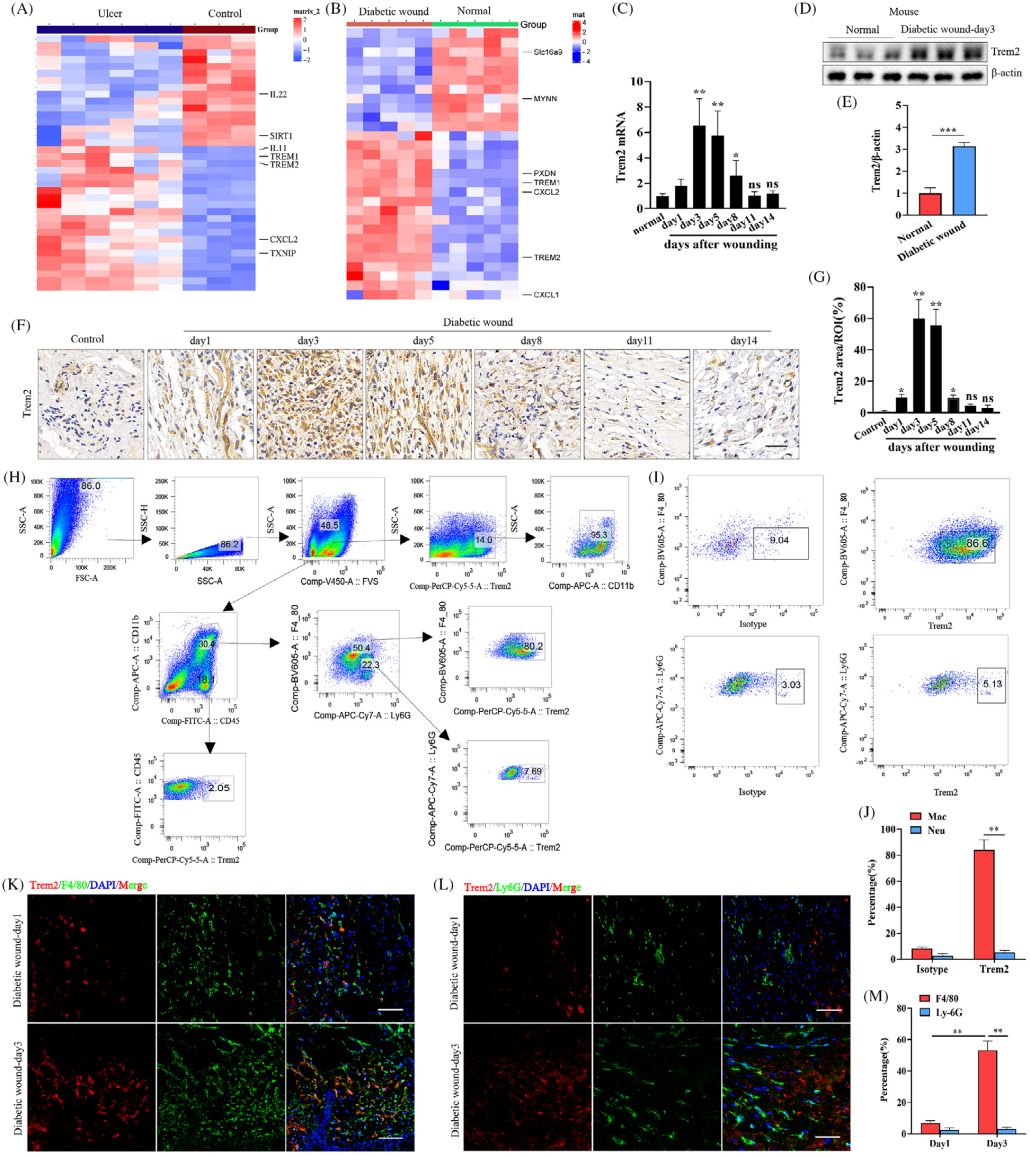
Trem2 levels were shown to be increased in diabetic ulcers and diabetic wound healing. (A) Heatmap of different gene expression patterns in human samples from control and diabetic ulcers. (B) Heatmap of different gene expression patterns in mouse samples of control and skin wounding. (C) Trem2 expression was examined via qPCR from skin wounding on days 1, 3, 5, 8, 11 and 14 post‐injury as well as in controls. *n* = 6 samples/group. (D) Western blot analysis of Trem2 levels in skin wounding on day 3 post‐injury and control. *n* = 5 samples/group. (E) Quantified data of immunoblotting band intensity in D. (F) Trem2 expression was analysed by immunohistochemical staining in skin tissues from skin wounding on days 1, 3, 5, 8, 11 and 14 post‐injury as well as in controls. *n* = 6 samples/group. (G) Quantified data of immunohistochemical staining intensity in F. (H) Flow cytometry gating strategy and analysis of the Trem2^+^ subpopulations of mouse skin immune cells at day 3 after diabetic wound injury (*n* = 4). (I) Flow cytometry analysis of the effects of Trem2 expression on gated CD11b^+^F4/80^+^ macrophages and CD11b^+^Ly‐6G^+^ neutrophils at day 3 after diabetic wound injury (*n* = 4). (J) Quantification of the flow cytometry data in I. (K, L) Representative dual‐immunofluorescence staining of Trem2 and F4/80 or Trem2 and Ly‐6G in skin tissues at day 1 and day 3 after injury. Scale bar is 100 µm. *n* = 6 samples/group. Scale bar is 100 µm. (M) Quantification of dual‐immunofluorescence staining intensity in K and L. *p < .05; **p < .01.

To initially explore genes that may be associated with diabetic wound healing, the GSE 80178 microarray dataset was downloaded from the Gene Expression Omnibus database. Trem2 was one of the DEGs significantly upregulated in the DFU relative to uninjured foot skin (Figure 1A). To extend these analyses to an in vivo experimental system, DFU model mice were established and gene expression profiles in diabetic wound tissues were analysed. This RNA‐seq approach revealed that skin wound generation in these animals was associated with significant *Trem2* upregulation (Figure 1B). Subsequent qPCR, immunohistochemistry staining and Western immunoblotting analyses demonstrated that while Trem2 expression levels in the skin at baseline were relatively low, it was markedly upregulated in response to cutaneous wounding in diabetic model mice, with maximum levels being evident on day 3 post‐wounding after which these levels gradually declined over the course of the healing process (Figure 1C−G). Next, we sought to identify the leukocytes that affect Trem2 expression after diabetic wound injury. Flow cytometry showed that almost all Trem2‐expressing cells in the skin tissues at day 3 after diabetic wound injury were CD11b^+^ myeloid cells; however, the lymphocytes did not express Trem2 (Figure 1H). Among the CD11b^+^ myeloid cells, nearly 80% of CD11b^+^F4/80^+^Ly6G macrophages expressed Trem2, whereas CD11b^+^Ly6G^+^F4/80^−^neutrophils made up only a minor portion of Trem2‐expressing leukocytes (Figure 1I,J). Dual immunofluorescent staining for Trem2 and either F4/80 or Ly6G in skin tissue samples from the diabetic model mice on days 1 and 3 post‐wounding revealed that Trem2 expression was specifically enriched in macrophages but not neutrophils in response to DFU model wound generation (Figure 1K−M). These results reveal that Trem2 may play a role in regulating the formation or healing of diabetic ulcers.

### 3.2 Macrophage‐specific loss of Trem2 expression impairs in vivo diabetic wound closure

**FIGURE 2:**
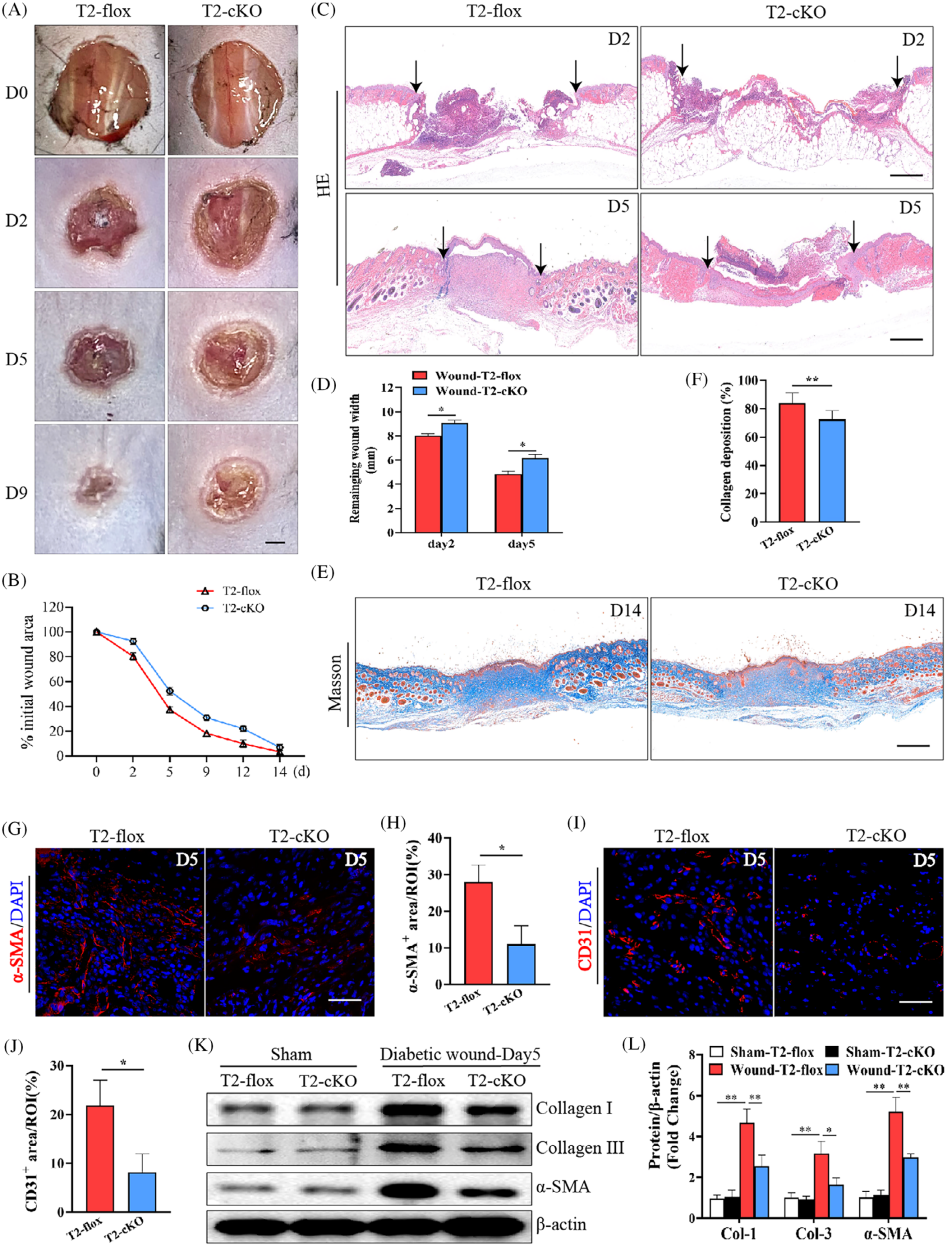
Trem2 knockout impaired wound healing in diabetic mice. (A) Trem2 knockout increased skin wound areas on the indicated days. *n* = 8 samples/group. (B) Quantification of wound area. Data are expressed as the percentage of the remaining area to the initial wound area. (C, D) Images of histological staining and quantification on day 2 and day 5 post‐injury. Scale bar is 100 µm. *n* = 8 samples/group. (E, F) Images of Masson staining and quantification on day 14 post‐injury. *n* = 8 samples/group. (G, H) Images of immunofluorescence staining of α‐SMA and quantification on day 5 post‐injury. Scale bar is 100 µm. *n* = 6 samples/group. (I, J) Images of immunofluorescence staining of CD31 and quantification on day 5 post‐injury. Scale bar is 100 µm. *n *= 6 samples/group. (K, L) The expression of α‐SMA, COL1 and COL3 was detected by WB on day 5 post‐wounding. *n* = 5 samples/group. *p < .05; **p < .01.

To explore the importance of macrophage Trem2 expression in the induction of wound healing, a diabetic wound model was established in T2‐cKO mice bearing a macrophage‐specific deletion of Trem2. Firstly, we confirmed the absence of Trem2^+^ cells in T2‐cKO mice by flow cytometry (Figure S4). Relative to T2‐flox control mice, these T2‐cKO animals did not exhibit any baseline skin defects (Figure S5). However, wound healing was notably impaired on days 2, 5 and 9 post‐wounding in these T2‐cKO animals (Figure 2A,B), with representative images and measurements of epithelial gap sizes indicating that wounds were significantly larger in T2‐cKO mice on days 2 and 5 relative to T2‐flox controls (Figure 2C,D). On day 14 post‐injury, Masson's trichrome staining analyses of collagen deposition revealed that T2‐cKO mice exhibited a significantly reduced collagen volume fraction compared to T2‐flox controls (Figure 2E,F), and immunofluorescent staining for α‐SMA and CD31 revealed poor tissue repair and angiogenic activity on day 5 post‐wounding in these T2‐cKO mice (Figure 2G−J). Western immunoblotting further suggested that the macrophage‐specific loss of Trem2 suppresses the expression of key repair‐related proteins on day 5 post‐wounding including α‐SMA, COL1 and COL3 (Figure 2K,L). Above all, these results reveal that the loss of macrophage Trem2 expression impairs the diabetic wound healing process in mice.

### 3.3 The macrophage‐specific deletion of Trem2 induces excessive inflammatory activity and oxidative stress in a murine model of diabetic wound healing

**FIGURE 3:**
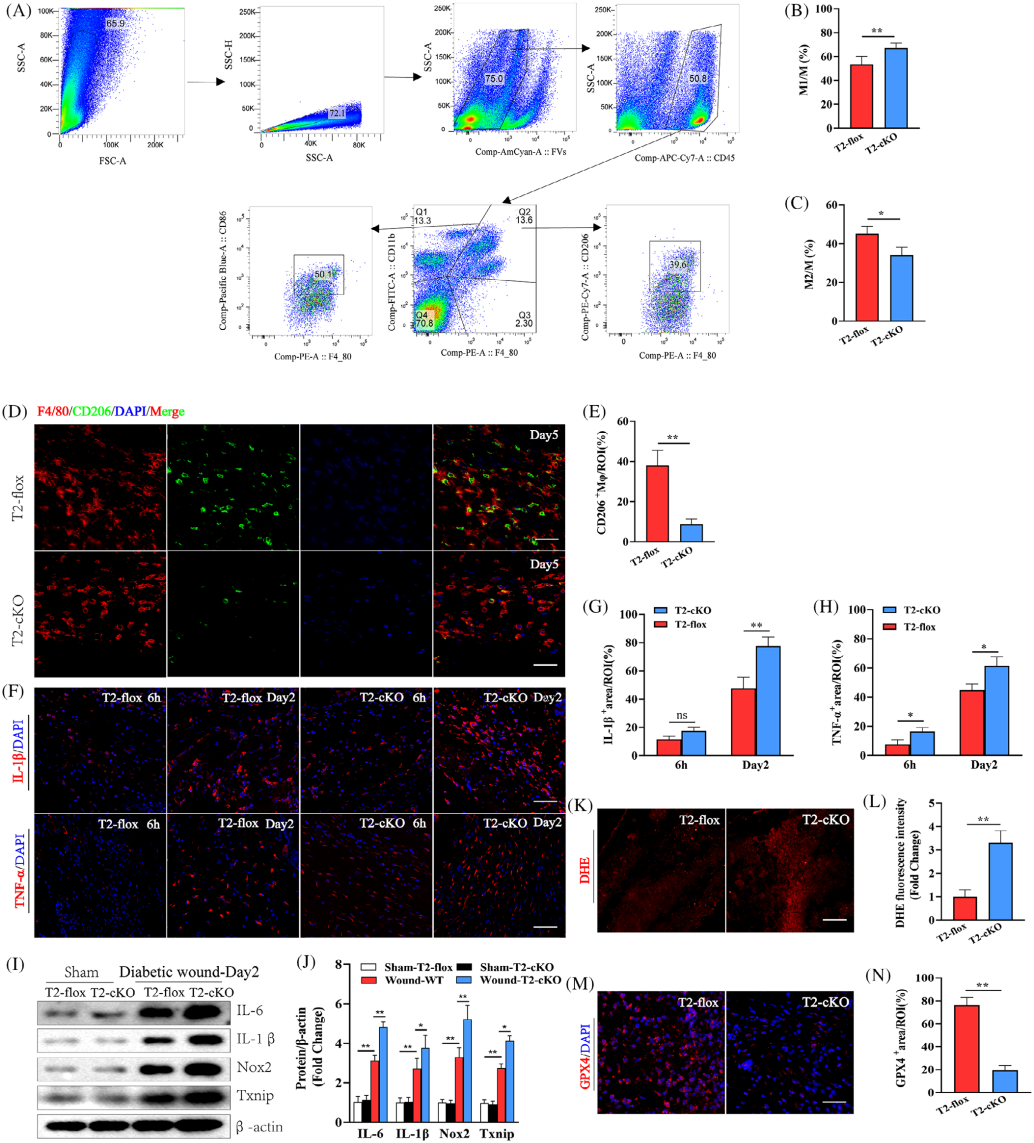
Attenuated inflammatory phenotype in wound injury macrophages with Trem2 loss‐of‐function. (A) Gating strategy for pro‐inflammatory macrophages (F4/80^+^CD11b^+^CD86^+^), and anti‐inflammatory macrophages (F4/80^+^CD11b^+^CD206^+^). (B, C) The numbers of pro‐inflammatory and anti‐inflammatory macrophages were determined in the skin tissues of WT and T2KO mice at day 2 after diabetic wound injury. (D, E) Images of immunofluorescence staining of CD206, F4/80 and quantification on day 5 post‐injury. Scale bar is 100 µm. *n* = 6 samples/group. (F−H) The expression of IL‐1β and TNF‐α was detected by immunofluorescence staining. Scale bar is 100 µm. *n *= 6 samples/group. (I, J) The expression of IL‐6, IL‐1β, Nox2 and Txnip was detected by WB on day 2 post‐wounding. *n *= 5 samples/group. (K, L) Representative images and quantitative analysis showing the levels of superoxide anions as measured by DHE staining. *n *= 5 samples/group. (M, N) Images of immunofluorescence staining of GPX4 and quantification on day 2 post‐injury. Scale bar is 100 µm. *n *= 6 samples/group. *p < .05; **p < .01.

The appropriate healing of diabetic wounds is highly dependent on an appropriate transition from an inflammatory to a proliferative state.^20^, ^21^ Next, we investigated macrophage phenotype in skin tissues at day 2 after diabetic wound injury using flow cytometry. The gating strategy used for the identification of pro‐inflammatory macrophages (F4/80^+^CD11b^+^CD86^+^) and anti‐inflammatory macrophages (F4/80^+^CD11b^+^CD206^+^) is shown in Figure 3A. The results showed that Trem2 deficiency led to enhanced recruitment of F4/80^+^CD11b^+^CD86^+^ pro‐inflammatory macrophages in the skin tissues and a significant reduction in the proportion of F4/80^+^CD11b^+^CD206^+^ anti‐inflammatory macrophages compared with Wild type (WT) mice at day 2 after diabetic wound injury (Figure 3B,C). Next, using immunofluorescent staining for the phenotypic marker CD206 to evaluate anti‐inflammatory macrophage abundance in the DFU mouse model system established above, a clear reduction in anti‐inflammatory macrophage levels was observed in T2‐cKO mice (Figure 3D,E). The healing of skin wounds can be severely negatively impacted by both excessive inflammation and oxidative stress.^22^ Accordingly, both inflammatory and oxidative stress responses were analysed via immunofluorescent staining in murine skin at 6 h and 2 days post‐wounding, revealing that the knockout of Trem2 can enhance IL‐1β and TNF‐α expression in macrophages (Figure 3F−H), with Western immunoblotting yielding similar results on day 2 post‐wounding (Figure 3I,J). A DHE assay was further employed to measure ROS levels, with T2‐cKO mouse samples containing significantly higher ROS levels relative to T2‐flox controls in this experimental setting on day 2 post‐wounding (Figure 3K,L). Consistently, the intensity of the fluorescent signal for the antioxidant enzyme glutathione peroxidase 4 (GPX4) was notably reduced in T2‐cKO mice as compared to T2‐flox controls on day 2 post‐wounding (Figure 3 M,N). These results demonstrate that the macrophage‐specific deletion of Trem2 significantly enhanced inflammatory responses, oxidative stress and ferroptotic cell death in these diabetic model mice following skin wounding.

### 3.4 IL‐4 binds directly to Trem2 protein

**FIGURE 4:**
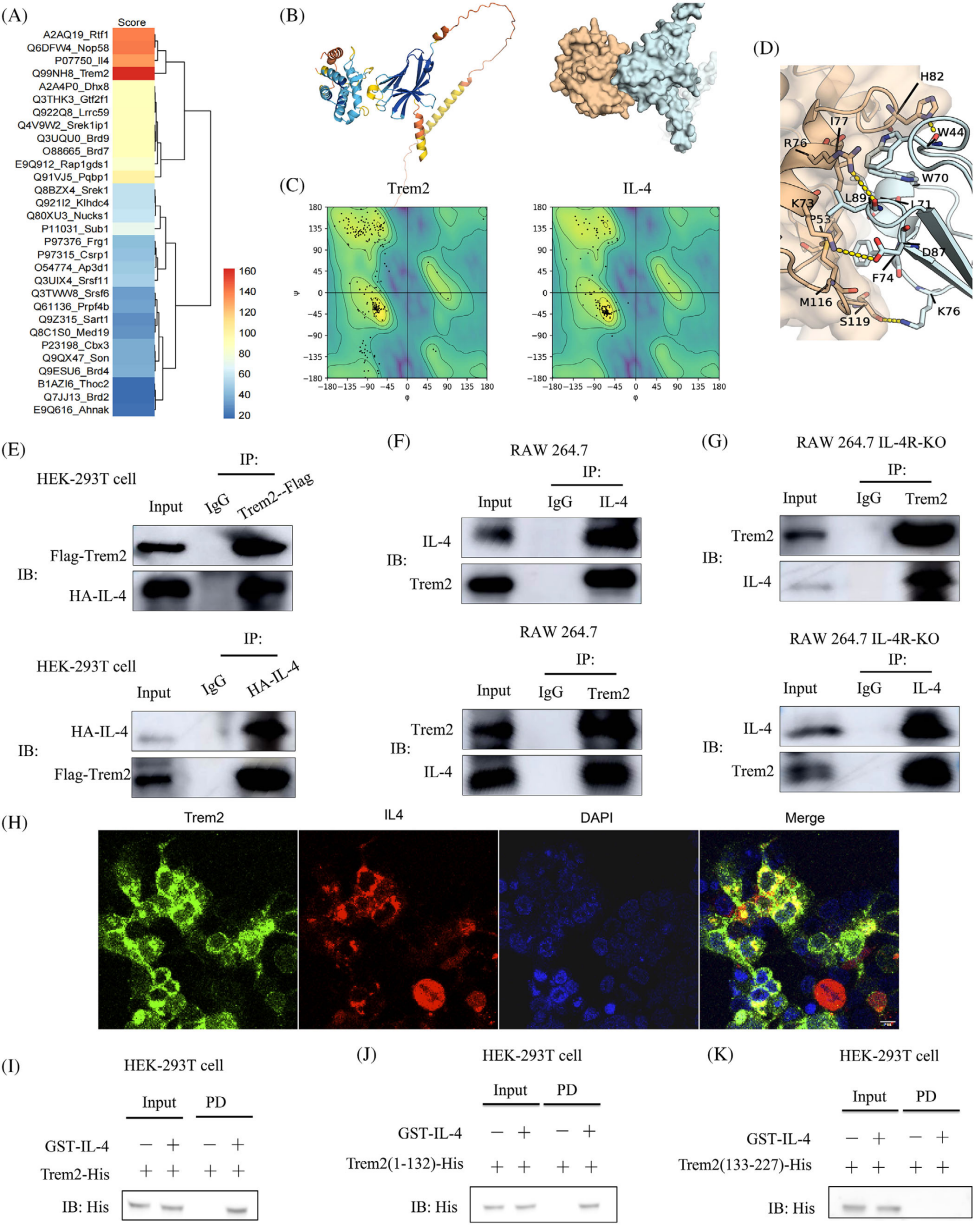
IL‐4 binds directly to Trem2 protein. (A) Heatmap of Trem2 target proteins in macrophages. (B) Left: Cartoon representation of the complex with colours indicating the pLDDT confidence scores from AlphaFold. Right: Surface‐binding model of IL‐4 with Trem2. (C) Ramachandran plot. (D) Details of the interaction between IL‐4 and Trem2. IL‐4 is shown in cyan and Trem2 in orange; residues in IL‐4 are coloured cyan, while residues in Trem2 are coloured orange; red dashes represent hydrogen bond interactions; blue dashes represent salt bridges. (E) Co‐immunoprecipitation of Trem2 and IL‐4 in HEK‐293T cells. (F, G) Co‐immunoprecipitation of endogenous Trem2 and IL‐4 in RAW 264.7 and RAW 264.7 IL‐4R‐KO. (H) Immunofluorescence analysis revealed that Trem2 co‐localized with IL‐4 on the macrophages. Scale bar: 100 µm. (I) Direct binding of GST‐IL‐4 to His‐Trem2 using GST pull‐down assay. (J, K) GST pull‐down assay examining interactions between GST‐fused IL‐4 and various His‐Trem2 protein fragments.

It has been reported that the phenotypic transition of macrophages is controlled by a series of inflammatory factors. We, therefore, searched for alternative Trem2 ligands in macrophages using mass spectrometry. The top 30 proteins identified as potential targets of Trem2 are listed in Figure 4A, showing the unexpected inclusion of IL‐4 with a high score. To determine the nature of the IL‐4/Trem2 interaction, we used AlphaFold‐multimer to predict the complex structure of IL4 and Trem2 (Figure 4B). Also, we conducted a Ramachandran plot to explore the distribution of the dihedral angles of individual residues from the predicted protein structures (Figure 4C). Furthermore, the detailed interface interactions between IL4 and Trem2 were displayed (Figure 4D). We further investigated the interaction between IL‐4 and Trem2 using co‐precipitation. HEK 293T were also transfected with Trem2 or IL‐4, showing that IL‐4 could interact with Trem2 in HEK‐293T cells (Figure 4E). Similar results were found in RAW 264.7 and RAW 264.7 IL‐4R‐KO cells (Figure 4F,G), confirming that IL‐4 binds to Trem2 is independent of IL‐4R. IF analysis showed that Trem2 co‐localized with IL‐4 on the macrophage (Figure 4H). We further constructed plasmids expressing the full‐length mouse Trem2 or IL‐4 sequences, and purified the Trem2 protein with an N‐terminal His tag and GST‐IL‐4 (Figure S6). GST pull‐down assays showed that His‐Trem2 binds to GST‐IL‐4 (Figure 4I). To determine the region of Trem2 responsible for the interaction with IL‐4, we created His‐tagged N‐terminal aa1−aa132 of Trem2 (NT‐aa1−132), His‐tagged NT aa133−227 of Trem2 (NT‐aa133−227) and His‐tagged full‐length Trem2 constructs, and expressed these proteins in HEK‐293T cells. GST‐tagged IL‐4 was found to associate with the full‐length or NT‐aa1‐132 fragment of Trem2 but not with the NT‐aa133‐227 fragment (Figure 4J,K). These results indicate that Trem2 is a non‐classical receptor of IL‐4 in macrophages.

### 3.5 Trem2 deletion in macrophages impairs dermal fibroblast functions due to the disruption of appropriate paracrine signalling

**FIGURE 5:**
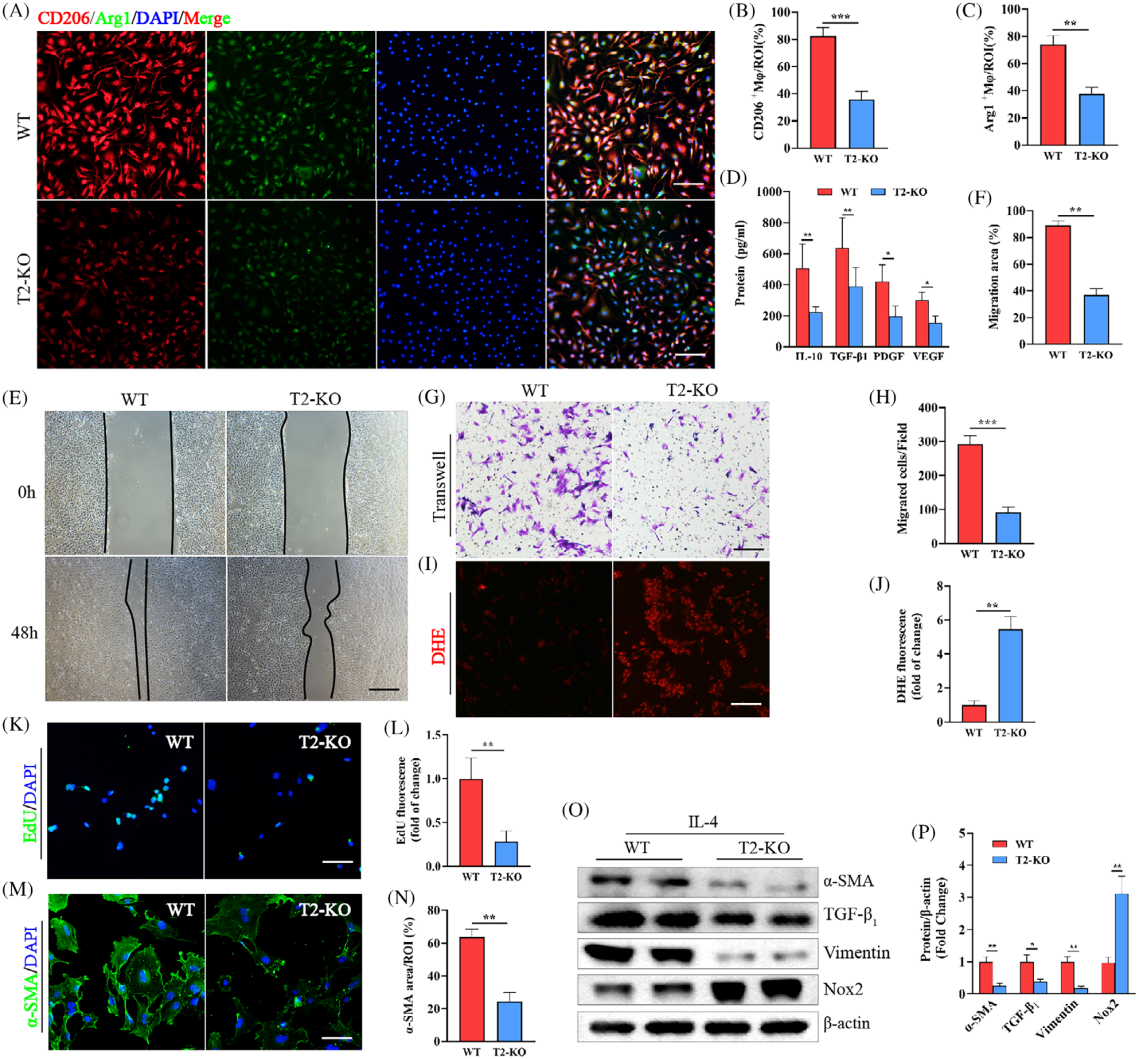
Trem2 was identified as a functional receptor mediating the effects of IL‐4 and Trem2 knockout in macrophages impaired skin fibroblasts function and promotes ROS levels. (A−C) Images of immunofluorescence staining of CD206, Arg1 in macrophage and quantification. Scale bar is 100 µm. *n *= 7 samples/group. (D) Quantification of the expression of IL‐10, TGF‐β1, PDGF and VEGF by Elisa assay. *n* = 7 samples/group. (E, F) Wound healing assay and quantification. *n* = 6 samples/group. (G, H) Transwell assays and quantification. *n *= 6 samples/group. (I, J) Representative images and quantitative analysis showing the levels of superoxide anions as measured by DHE staining. *n* = 6 samples/group. (K, L) Representative images and quantitative of EdU staining. *n *= 6 samples/group. (M, N) Representative immunofluorescence images of α‐SMA expression in skin fibroblasts and quantification. Scale bar is 100 µm. *n *= 6 samples/group. (O, P) α‐SMA, vimentin and TGFβ1 and Nox2 expression levels were measured by Western blotting, and the expression levels in the different groups were quantified. *n* = 5 samples/group. *p < .05; **p < .01; ***p < .001.

To explore the direct role that IL‐4/Trem2 plays as a regulator of pro‐inflammatory/anti‐inflammatory phenotypic differentiation in macrophages, BMDMs were harvested from WT or Trem2‐KO mice and treated with IL‐4. Subsequent immunofluorescent staining revealed a decrease in the expression of the anti‐inflammatory phenotypic marker proteins Arg1 and CD206 on macrophages from Trem2‐KO mice relative to those from WT controls (Figure 5A−C). Moreover, the loss of Trem2 expression was associated with a drop in the levels of secreted proteins associated with tissue repair including IL‐10, TGF‐β1, PDGF and VEGF, as measured by ELISA (Figure 5D). These data findings suggest that Trem2 is capable of directly driving the transformation of macrophages towards the anti‐inflammatory phenotype.

To examine the crosstalk between anti‐inflammatory macrophages and dermal fibroblasts, a co‐culture experimental system was established. This approach revealed that the macrophage‐specific knockout of Trem2 suppressed the migration of these fibroblasts in wound healing and transwell experiments (Figure 5E−H), inhibited their proliferation and contributed to the elevation of ROS levels within these dermal fibroblasts (Figure 5I−L). This loss of Trem2 expression was also associated with significant decreases in α‐SMA, vimentin and TGF‐β1 protein levels within dermal fibroblasts, whereas Nox2 expression was increased in these cells (Figure 5M−P). These results support the ability of macrophage Trem2 expression to enhance the function of dermal fibroblasts through a paracrine signalling effect.

### 3.6 Transcriptomic sequencing‐based identification of the signalling pathways regulated by Trem2

**FIGURE 6:**
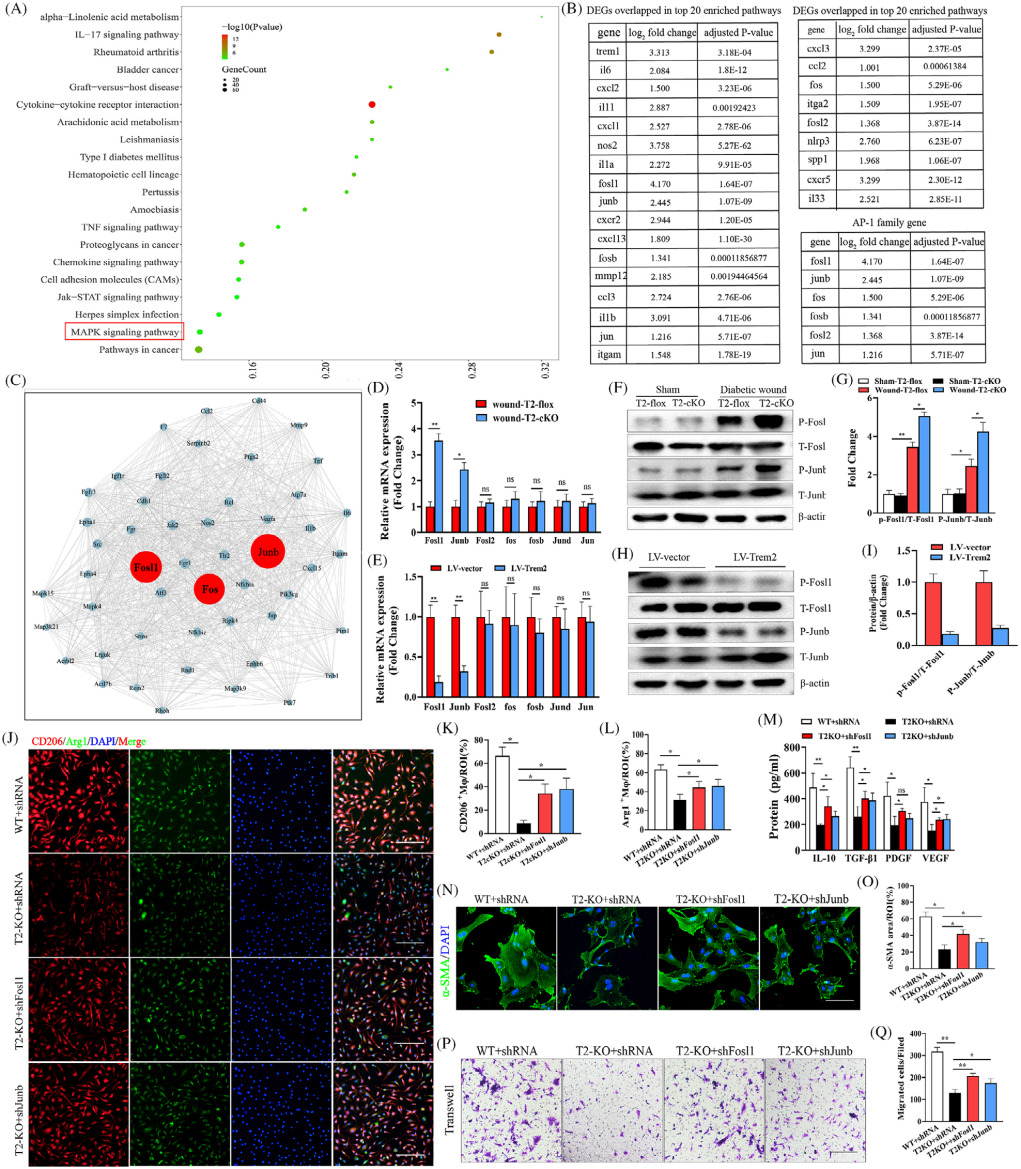
RNA sequencing highlighting of signalling pathways and target molecules modulated by Trem2. (A) Gene ontology (GO) analysis. (B) Changes in the expression of genes belonging to the AP‐1 family, shown by transcriptomic profiling data. (C) Protein−protein interaction network. (D) mRNA expression of AP‐1 family members, including Fosl1, c‐Fos, Junb, Fosl2, Fos, Fosb, Jund and Jun in wound issues. *n *= 5 samples/group. (E, F) The expression of p‐Fosl1, Fosl1, P‐Junb and Junb is shown by WB in wound issues and their quantification. *n* = 5 samples/group. (G) mRNA expression of AP‐1 family members, including Fosl1, c‐Fos, Junb, Fosl2, Fos, Fosb, Jund and Jun in macrophages. *n* = 5 samples/group. (H, I) The expression of p‐Fosl1, Fosl1, P‐Junb and Junb is shown by WB in macrophages and their quantification. *n* = 5 samples/group. (J−L) Immunofluorescence staining of CD206 and Arg1 in macrophages and their quantification. Scale bar is 100 µm. *n *= 5 samples/group. (M) Quantification of the expression of IL‐10, TGF‐β1, PDGF and VEGF by ELISA. *n *= 5 samples/group. (N, O) Representative immunofluorescence images of α‐SMA expression in skin fibroblasts and its quantification. Scale bar is 100 µm. *n *= 5 samples/group. (P, Q) Transwell assays and quantification. *n* = 5 samples/group. *p < .05; **p < .01.

To explore the mechanistic processes through which Trem2 functions as a regulator of diabetic wound healing, injured skin tissue samples from T2‐cKO and T2‐flox mice were collected for transcriptomic analysis. This RNA‐seq approach ultimately identified 687 and 596 DEGs that were significantly upregulated and downregulated, respectively, in T2‐cKO mice relative to T2‐flox controls on day 3 after wounding (Supplementary Material 7A). Functional clustering analyses revealed that many of these DEGs were associated with key inflammation‐ and oxidative stress‐related pathways including the IL‐17, TNF, JAK‐STAT and MAPK signalling pathways (Figure 6A). Further analysis of the DEGs overlapping among these top 20 pathways revealed that the majority (including *IL‐6*, *IL‐1a*, *IL‐11*, *Fosl1*, *Junb*, *Fos*, *Trem1*, *Ccl2*, *Cxcl2*, *Cxcl3*, *Cxcl10*, *Ccl3* and *Atgam*) serve as pro‐inflammatory mediators that were upregulated in T2‐cKO mice following skin wounding (Figure 6B).

When these DEGs were subjected to a protein−protein interaction network analysis, a close relationship was found between diabetic wound healing and the activator protein 1 (AP‐1) family members Fosl1 and Junb (Figure 6C). The AP‐1 transcription factor complex is comprised of different Fos and Jun family proteins, and serves as an important driver of inflammatory response induction.^23^, ^24^ Accordingly, these AP‐1 family proteins were selected as targets of interest for further experimental evaluation. A qPCR analysis revealed that these members of the AP‐1 transcription complex were significantly upregulated in skin tissue samples from T2‐cKO mice after wounding (Figure 6D). A luciferase reporter assay further demonstrated that the level of AP‐1 activity in T2‐cKO mice was significantly increased as compared to T2‐flox control animals (Supplementary Material S7B), with Western immunoblotting yielding similar results (Figure 6E,F). To test the ability of exogenous Trem2 to suppress Fosl1 and Junb expression and activity, BMDMs from WT mice were further stimulated with 10 ng/mL M‐CSF, after which the resultant M0 macrophages were transduced with the LV‐Trem2 or LV‐GFP vectors. Subsequent qPCR analysis highlighted a clear drop in Fosl1 and Junb levels following the overexpression of Trem2 (Figure 6G), with a luciferase reporter assay also demonstrating a consistent decline in AP‐1 activity following such overexpression (Supplementary Material S7C), Western immunoblotting yielding similar results in vitro (Figure 6H,I). These findings thus provide clear evidence that Trem2 can target AP‐1 and function as an inhibitor of macrophage‐driven inflammatory activity in the context of diabetic wound healing.

### 3.7 Fosl1 and Junb knockdown reverses the impact of the loss of Trem2 expression on in vitro and in vivo diabetic wound healing

To explore the importance of Trem2‐mediated AP‐1 regulation in the wound healing process, Fosl and Junb were next knocked down in cells of tissues using lentivirus constructs encoding shRNAs specific for these proteins. When BMDMs from WT and Trem2‐KO mice were infected with these lentiviral vectors and stimulated with IL‐4, subsequent IF staining showed that the silencing of Fosl1 and Junb was sufficient to alleviate the adverse impact of Trem2 deletion of the expression of the anti‐inflammatory macrophage marker proteins Arg1 and CD206 (Figure 6J−L). This silencing of Fosl1 and Junb additionally weakens the observed downregulation of the tissue repair‐associated proteins IL‐10, TGF‐β1, PDGF and VEGF in the context of Trem2 knockout (Figure 6M).

To expand on these findings, WT or Trem2‐KO macrophages were transfected with the shFosl1 or shJunb vectors prior to co‐culture with dermal fibroblasts. This approach revealed that knocking down Fosl1 and Junb improved the suppression of fibroblast activation (Figure 6N,O) and migration (Figure 6P,Q) observed when Trem2 was knocked out. The in vivo knockdown of Fosl1 and Junb was further achieved in the skin tissue of experimental mice by injecting the prepared shRNA‐encoding virus particles into the skin after injury. The knockdown of Fosl1 and Junb in these mice resulted in the enhancement of wound healing, as evidenced by a reduction in the epithelial gap and improved collagen alignment relative to those in control T2‐cKO mice on day 6 post‐wounding (Supplementary Material S8A−E). IF staining also indicated that the knockdown of these two AP‐1 family proteins improved the inflammation and ROS production that were otherwise observed on day 3 post‐injury in the skin of Trem2‐KO mice (Supplementary Material S8F−I). Staining for α‐SMA and CD31 also yielded similar results, demonstrating that knocking down Fosl1 and Junb increased the protein expression levels observed in Trem2‐KO mice (Supplementary Material S8J−M). Together, these findings indicate that the loss of Trem2 expression can impair wound healing at least in part via enhancing the expression of Fosl1 and Junb.

### 3.8 The knockout of Trem2 promotes AP‐1 expression via the MAPK signalling pathway

**FIGURE 7:**
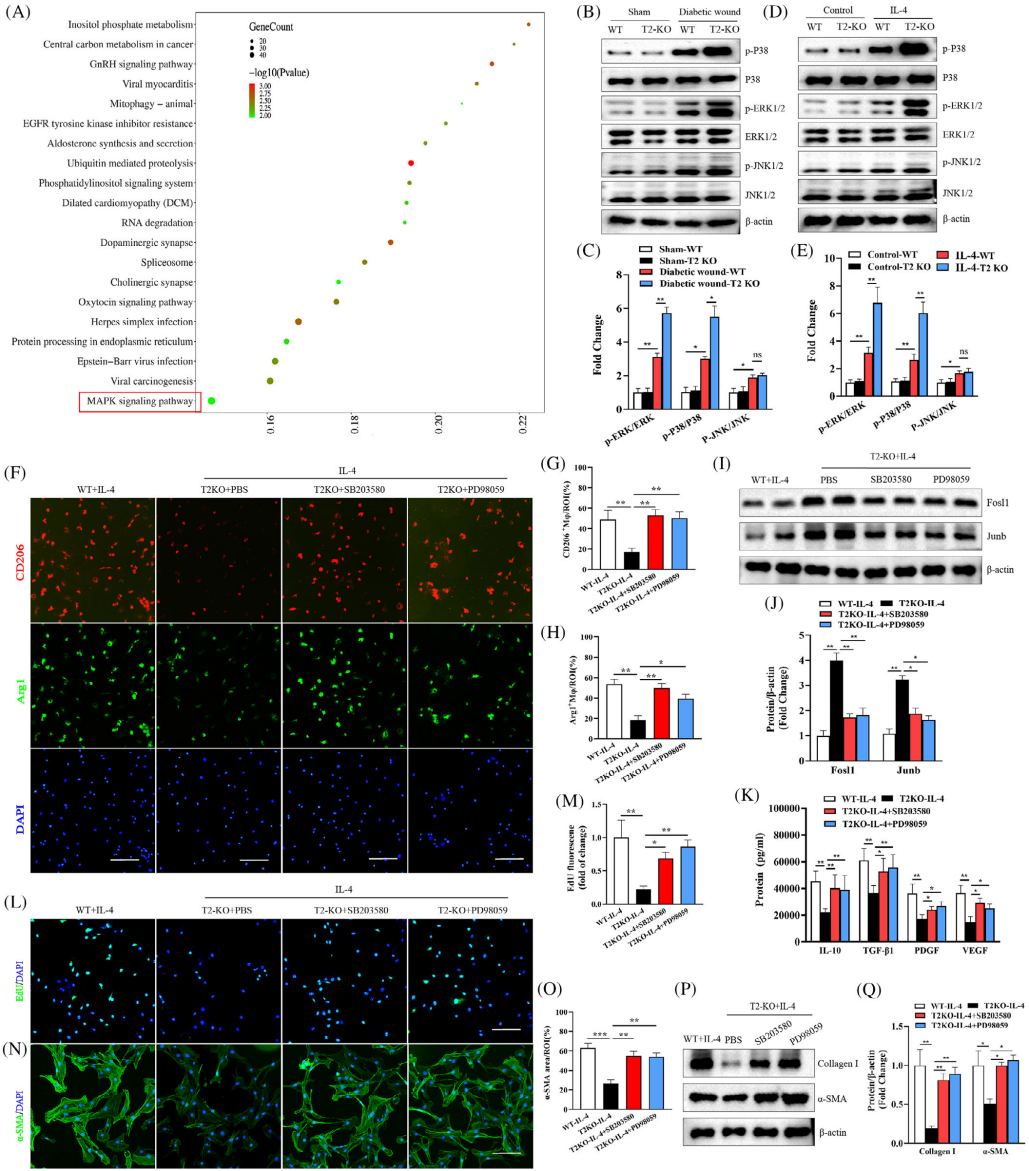
Trem2 knockout promoted the expression of AP‐1 by targeting MAPK signalling. (A) Gene ontology (GO) analysis results of differentially expressed genes. (B−E) The expression of ERK, JNK and P38 was detected by WB and their quantification. *n *= 5 samples/group. (F−H) Images of immunofluorescence staining of CD206, Arg1 in macrophages and their quantification. Scale bar is 100 µm. *n *= 6 samples/group. (I, J) The expression of Fosl1 and Junb was detected by WB. *n* = 5 samples/group. (K) Quantification of the expression of IL‐10, TGF‐β1, PDGF and VEGF by Elisa assay. *n *= 7 samples/group. (L, M) Representative images and quantitative of EdU staining. *n* = 6 samples/group. (N, O) Representative immunofluorescence images of α‐SMA expression in skin fibroblasts and quantification. Scale bar is 100 µm. *n *= 6 samples/group. (P, Q) The expression of collagen I and α‐SMA was detected by WB. *n* = 5 samples/group. *p < .05; **p < .01.

To more fully explore how Trem2 regulates AP‐1 activity, we stimulated WT macrophages and T2‐KO macrophages with IL‐4 and performed transcriptomic analysis of the cell samples. Functional cluster analysis revealed that Trem2 is also involved in the regulation of MAPK signalling at the cellular level (Figure 7A). This was consistent with the tissue sequencing results in Figure 6A. The modulation of Trem2 expression in vitro has previously been reported to have a significant impact on MAPK signaling.^19^, ^20^ In this study, Western immunoblotting revealed that the knockout of Trem2 enhanced p‐ERK and p38‐expression levels compared with the WT group in vitro and in vivo. However, the p‐JNK expression levels was no significant difference between the WT group and the T2‐KO WT group in diabetic mice (Figure 7B−E). Treatment with the ERK inhibitor SB203580 or the p38 inhibitor PD98059 partially reversed the negative effects of Trem2 deletion in vitro, increased the expression of both CD206 and Arg1 (Figure 7F−H) and  reduced the expression of both Fosl1 and Junb (Figure 7I,J). SB203580 and PD98059 blocked the effect of Trem2 knockout on the reduced secretion of IL‐10, TGF‐β1, PDGF and VEGF, as measured via ELISA, relative to the Trem2‐KO^+^IL‐4 treatment group (Figure 7K).

Cells treated with SB203580 and PD98059 were further used in a co‐culture analysis of the crosstalk between anti‐inflammatory macrophages and dermal fibroblasts. In this experimental setting, a significant increase in the proliferation (Figure 7L,M) and phenotypic transformation (Figure 7N,O) of dermal fibroblasts was observed following SB203580 and PD98059 treatment, with Western immunoblotting yielding similar results (Figure 7P,Q). These findings indicate that the loss of Trem2 can promote AP‐1 expression and dermal fibroblast phenotypic changes at least in part through the regulation of the MAPK signalling pathway.

### 3.9 Trem2 knockout impairs beneficial crosstalk between macrophages and dermal fibroblasts through the MAPK/AP‐1 pathway‐mediated upregulation of Lif

**FIGURE 8:**
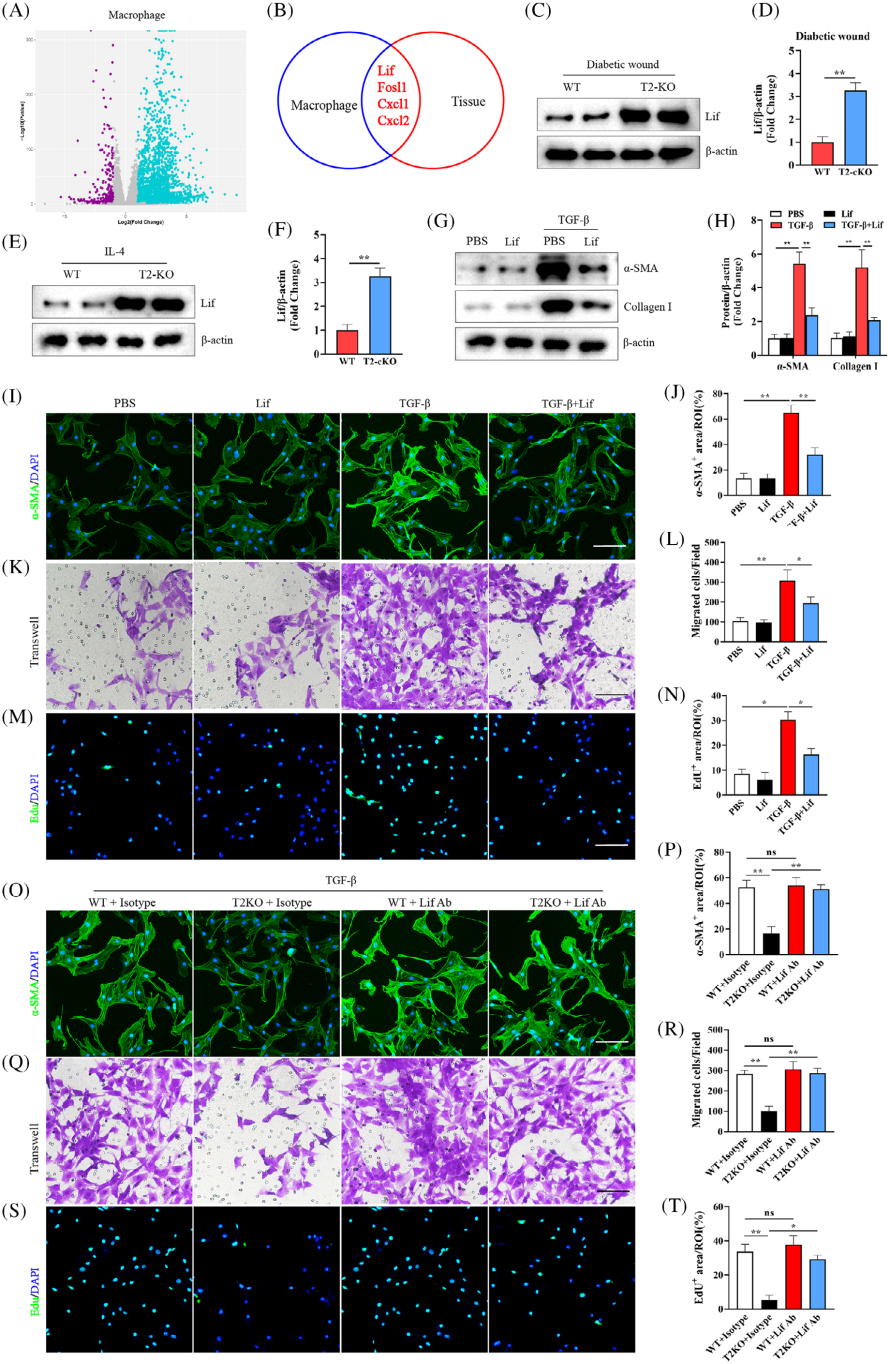
Trem2 deficiency impaired wound healing and fibroblast activation through Lif regulation.(A)Volcano plot analysis results of differentially expressed genes in macrophage. (B) Venn diagram of RNA sequencing data of macrophage (WT^+^IL‐4 vs. Trem2^−^/^−+^IL‐4) and tissue (T2‐flox vs. T2‐cKO). (C−F) The expression of Lif was detected by WB, and the expression levels in the different groups were quantified. *n *= 5 samples/group. (G, H) The expression of α‐SMA and Collagen I was detected by WB, and the expression levels in the different groups were quantified. *n *= 5 samples/group. (I, J and O, P) Representative immunofluorescence images of α‐SMA expression in skin fibroblasts and quantification. Scale bar is 100 µm. *n *= 6 samples/group. (K, L and Q, R) Transwell assays and quantification. Scale bar is 100 µm. *n* = 6 samples/group. (M, N and S, T) Representative images and quantitative of EdU staining. Scale bar is 100 µm. *n *= 6 samples/group. *p < .05; **p < .01.

To clarify the protective mechanisms through which macrophage‐derived Trem2 supports the diabetic wound healing process, macrophages were next used for transcriptional profiling. BMDMs from WT and Trem2‐KO mice were stimulated with IL‐4, and 645 and 511 DEGs were, respectively, found to be significantly upregulated and downregulated in Trem2‐KO cells relative to WT controls (Figure 8A). Several DEGs were found to be significantly enriched in both our macrophage (WT^+^IL‐4 vs. Trem2‐KO^+^IL‐4) and tissue (T2‐flox vs. T2‐cKO) datasets, including *lif*, *Fosl1*, *Cxcl1* and *Cxcl2* (Figure 8B), with changes in *lif* expression being successfully validated both in vitro and in vivo (Figure 8C−F). The ability of Trem2 to regulate the expression of Lif via the MAPK/AP‐1 signalling axis was then explored using ERK inhibitors, p38 inhibitors and shRNAs specific for Fosl1 and Junb. Western immunoblotting revealed that the knockout of Trem2 significantly promoted Lif upregulation, while these changes were reversed by inhibition of the activity of ERK and P38 and knocking down Fosl1 and Junb (Figure S9).

Lif functions as an important inhibitor of collagen expression and tissue fibrosis.^25^, ^26^ To test its functional role in the context of Trem2‐mediated regulation of diabetic wound healing, the impacts of Lif on dermal fibroblast proliferative, migratory and activation phenotypes were next evaluated. Briefly, dermal fibroblasts from WT mice were treated for 24 h with TGF‐β with or without Lif (1000 U/mL). Western immunoblotting demonstrated that Lif stimulation significantly inhibited the TGF‐β‐driven upregulation of α‐SMA and collagen I (Figure 8G,H). In this setting, Lif treatment was sufficient to suppress fibroblast proliferation, migration and phenotypic transformation as compared to control fibroblasts only treated with TGF‐β1 (Figure 8I−N). To explore the ability of Trem2 deletion to suppress the activation of fibroblasts in response to TGF‐β via the regulation of Lif, BMDMs were isolated from WT and Trem2‐KO mice and stimulated with IL‐4, after which the conditioned media from these cells was used to treat dermal fibroblasts together with TGF‐β. Treatment with a Lif‐neutralizing antibody reversed the suppression of fibroblast activation, migration and proliferation observed when these cells were treated with media from the Trem2‐KO^+^IL‐4 group (Figure 8O−T). These data thus indicate that the deletion of Trem2 interferes with diabetic wound healing in part by controlling the expression of Lif.

### 3.10 Administering soluble Trem2 enhances diabetic wound healing

**FIGURE 9:**
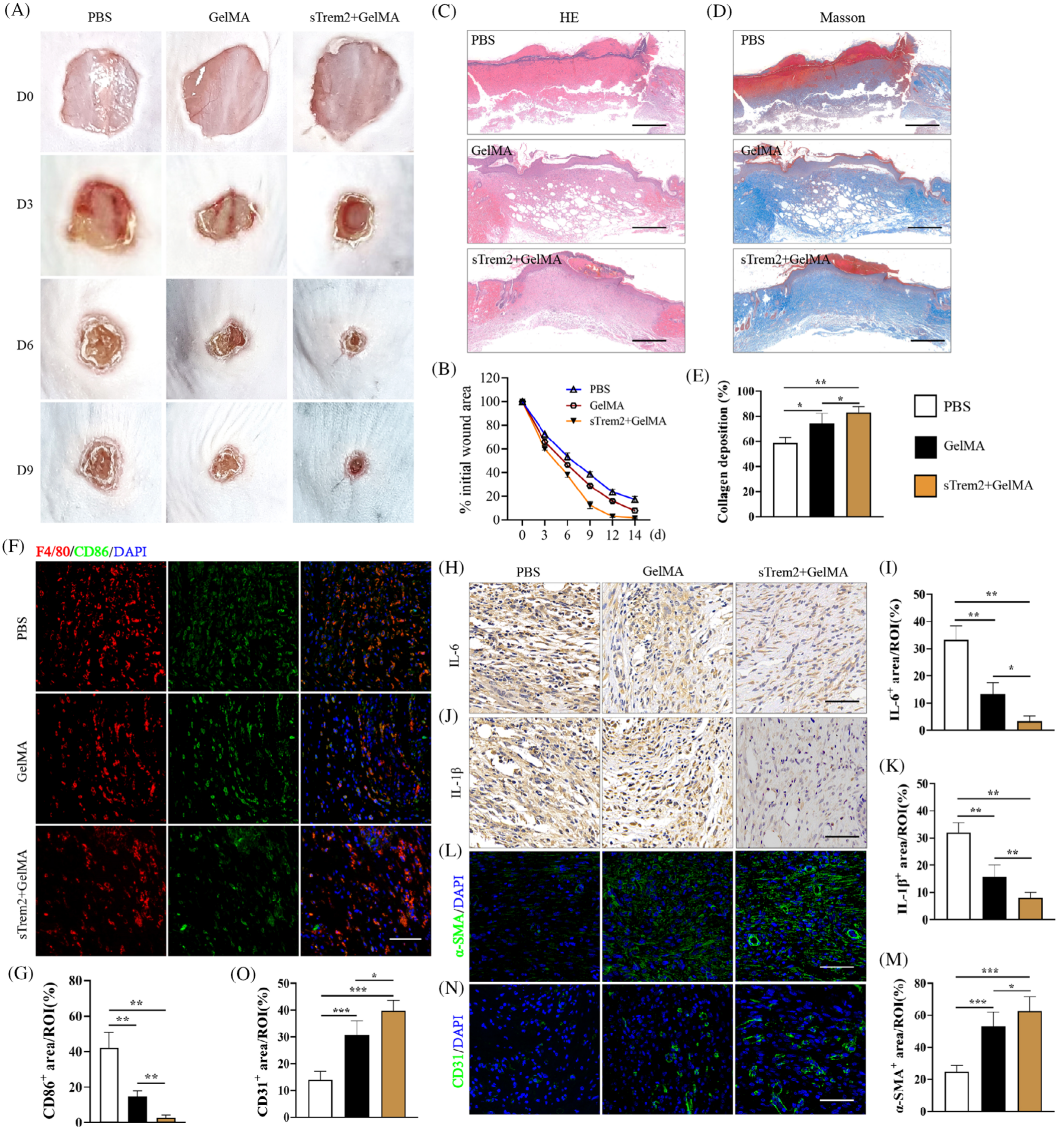
Effect of the injection of soluble Trem2 (sTrem2) on the wound healing in diabetic mice. (A) sTrem2 treatment decreased skin wound areas on the indicated days. *n* = 8 samples/group. (B) Quantification of wound area. Data are expressed as the percentage of the remaining area to the initial wound area. (C) Images of histological staining on day 6 post‐injury. Scale bar is 100 µm. *n *= 8 samples/group. (D, E) Images of Masson staining and quantification on day 14 post‐injury. *n *= 8 samples/group. (F, G) Images of immunofluorescence staining of CD86 and quantification on day 3 post‐injury. Scale bar is 100 µm. *n* = 6 samples/group. (H−K) Images of immunohistochemical staining of IL‐6, IL‐1β and quantification on day 3 post‐injury. *n *= 6 samples/group. (L, M) Images of immunofluorescence staining of α‐SMA and quantification on day 6 post‐injury. Scale bar is 100 µm. *n *= 6 samples/group. (N, O) Images of immunofluorescence staining of CD31 and quantification on day 6 post‐injury. Scale bar is 100 µm. *n* = 6 samples/group. *p < .05; **p < .01.

To test the impact of therapeutic sTrem2 administration on the in vivo diabetic wound healing process, sTrem2 was mixed with an injectable gelatin hydrogel as in prior reports.^27^ Next, we compared the changes in the wound closure and inflammatory activity among three groups of mice with diabetic wound:PBS group, GelMA alone group and sTrem2 was mixed with GelMA group (sTrem2‐GelMA). The administration of this sTrem2‐GelMA was associated with the significant acceleration of wound healing on days 3, 6 and 9 post‐injury relative to PBS group and GelMA alone group (Figure 9A,B), with a corresponding reduction in the measured epithelial gap size on day 6 (Figure 9C). Treatment with sTrem2‐GelMA also increased the observed collagen volume fraction during the healing process as detected via Masson's trichrome staining (Figure 9D,E). Relative to vehicle‐treated mice, those treated with sTrem2‐GelMA also exhibited a significantly decreased CD86^+^ cell proportion on day 3 after injury (Figure 9F,G). Immunohistochemistry (IHC) staining revealed a significant decline in IL‐6 and IL‐1β levels in the wounds of sTrem2‐GelMA‐treated mice (Figure 9H−K), while IF staining for α‐SMA and CD31 confirmed the ability of this sTrem2‐GelMA preparation to promote tissue repair and angiogenesis on day 6 post‐injury (Figure 9L−O). These results thus confirmed the ability of sTrem2 treatment to promote enhanced in vivo diabetic wound healing.

## 4 DISCUSSION

Macrophages are vital mediators of tissue repair and regeneration after wounding.^6^ The expression of the transmembrane receptor protein Trem2 is largely restricted to the myeloid compartment wherein it acts as a key regulator of macrophage‐related immune activities. No studies to date, however, have explored the role that Trem2 plays in the context of diabetic wound healing of the underlying molecular mechanisms responsible for such activity. This study is the first to our knowledge to have demonstrated that Trem2 is upregulated in a mouse model of diabetic wounding, with peak expression on day 3 after wound induction followed by gradual downregulation over the course of the healing process. The experiments conducted herein further revealed that while the macrophage‐specific deletion of Trem2 did not induce any baseline skin defects in these mice, they exhibited more robust inflammatory responses and impaired wound healing as compared to WT control animals post‐injury. Moreover, the frequency of anti‐inflammatory macrophages was decreased in these T2‐cKO mice after wounding, supporting a potential role for Trem2 as a functional regulator of the interconversion between pro‐inflammatory and anti‐inflammatory macrophage populations.

Several inflammatory cytokines regulate the phenotypic differentiation of macrophages, with IFN‐γ and IL‐4, respectively, promoting the differentiation of pro‐inflammatory and anti‐inflammatory macrophages.^28^, ^29^ The macrophage‐specific loss of Trem2 in the mice in this study was associated with a pronounced drop in the frequency of anti‐inflammatory macrophages, and Trem2 was further found to directly promote the anti‐inflammatory differentiation of murine macrophages in vitro. These findings are consistent with a model wherein the specific deletion of Trem2 in macrophages contributes to the induction of more severe inflammation and oxidative stress during the skin wound healing process. Trem2 may thus also serve as a key regulator of these inflammatory responses. Thus, further studies will be necessary to clarify the direct involvement of Trem2 in these inflammatory signalling pathways and to test its potential ability to interact with the IFN‐γ or IL‐4 receptors or downstream components in their signal transduction cascades.

Several studies have indicated that interactions with different ligands can modulate the strength and direction of Trem2 signalling differently.^30^ Trem2 has been shown to bind directly to mAβ42, thus blocking Aβ42 polymerization to inhibit the development of AD.^31^ Xie et al. found that IL‐34 binds directly to Trem2 to inhibit acute myeloid leukaemia in mice.^32^ We further investigated the molecular mechanisms underlying Trem2−ligand interactions using mass spectrometry. Our data showed that IL‐4 induced anti‐inflammatory macrophage phenotype through binding directly to Trem2. With IL‐4 stimulation, Trem2 deletion promoted pro‐inflammatory macrophage phenotype and impaired the functions of dermal fibroblasts. The results verified that Trem2 may act as a non‐classical receptor of IL‐4 to promote diabetic wound healing.

A comparison of the transcriptomic profiles of wounded skin tissue samples from Trem2‐flox and Trem2‐KO mice herein revealed that inflammation‐related gene signatures were more clearly evident in Trem2‐cKO mice as compared to Trem2‐flox controls, potentially contributing to the downstream induction of pro‐inflammatory signalling activity. Notably, pronounced AP‐1 upregulation and MAPK signalling activity were observed when Trem2 was knocked out in macrophages. The AP‐1 transcription factor is formed by different members of the Fos and Jun protein families, and serves as an essential promoter of cytokine expression in a range of inflammatory diseases including psoriatic arthritis, psoriasis and rheumatoid arthritis.^33^, ^34^ AP‐1 is also a target of several different anti‐inflammatory and antioxidant compounds.^35^ These findings thus align well with prior research results.

In this analysis, inhibiting Fosl1 and Junb was found to be sufficient to improve the effects that the loss of Trem2 had on in vitro and in vivo wound healing. This study is thus the first to demonstrate that wound healing can be impaired by Trem2 deficiency owing to the consequent upregulation of Fosl1 and Junb. MAPK signalling is also critically important to the differentiations of macrophages,^36^, ^37^ with the activation of the p38 and ERK1/2 components of the MAPK pathway favouring pro‐inflammatory differentiation. In the context of Parkinson's disease, Trem2 has been shown to negatively regulate MAPK signalling and thereby alleviate neuroinflammatory activity.^38^ Zhang et al. have further demonstrated that neuroinflammation can be regulated by the Trem2–p38 MAPK signalling axis in the context of chronic cerebral hypoperfusion and DM.^39^ In the present study, the loss of Trem2 expression resulted in enhanced p38 and ERK‐mediated MAPK signalling in lieu of signalling mediated by JNK, with treatment with ERK and p38 inhibitors (SB203580 and PD98059, respectively) being sufficient to change the effects that the loss of Trem2 had on Fosl1 and Junb expression. The loss of Trem2 expression can thus promote AP‐1 expression and activity while driving dermal fibroblast phenotypic transformation through the targeting of MAPK signalling, consistent with a role for Trem2 as a negative regulator of MAPK signalling and AP‐1 activation.

**FIGURE 10:**
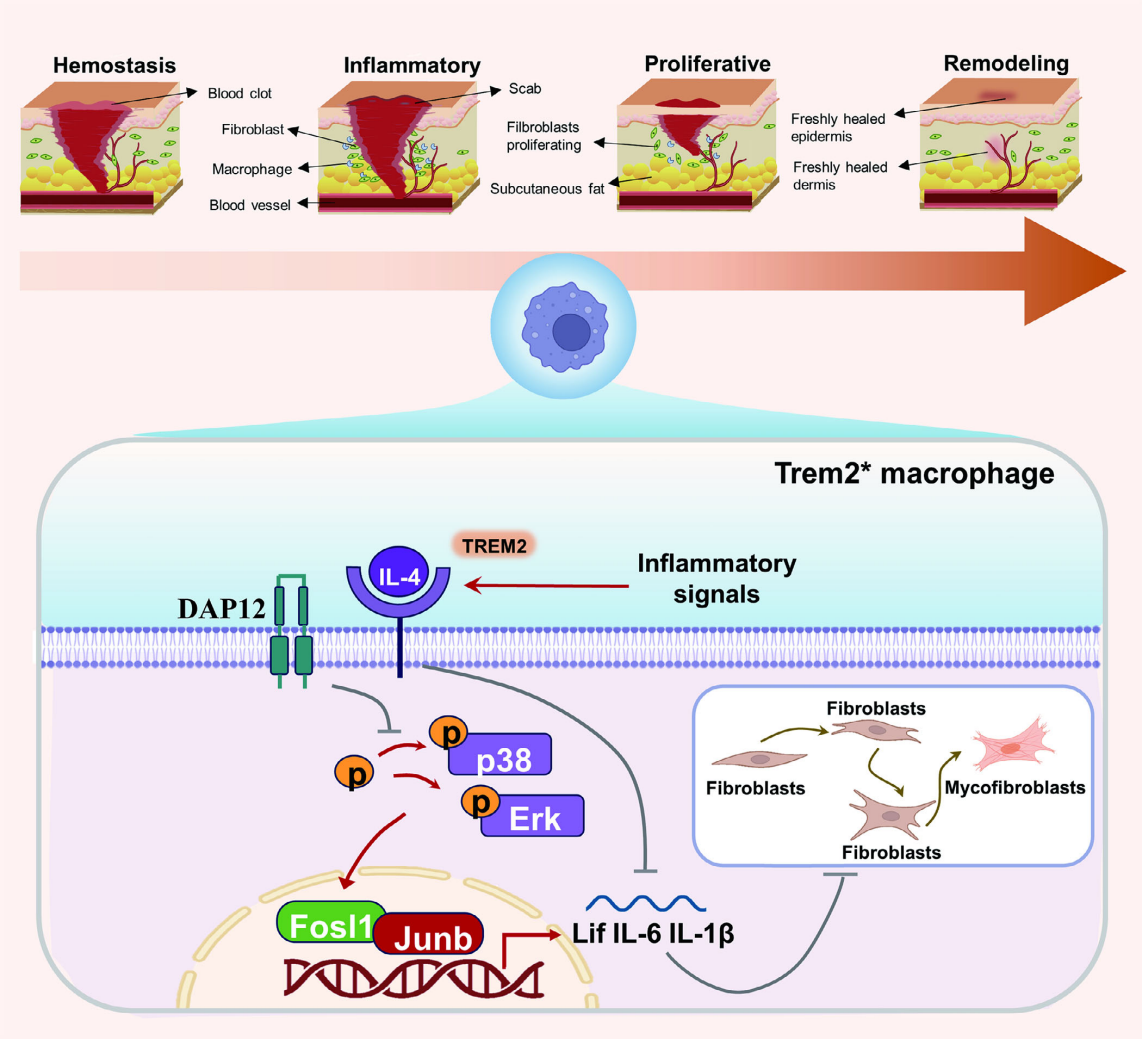
A working model of macrophage‐fibroblast crosstalk by the IL‐4/Trem2/MAPK/AP‐1/Lif signalling in diabetic wound healing.

Wound healing is an inherently intricate process that is further complicated by diabetes and relies on interactions between macrophages, dermal fibroblasts and other cell types.^40^, ^41^ The present results suggest that macrophages that lack Trem2 expression can suppress dermal fibroblast proliferative activity and phenotypic transformation, potentially by inducing the secretion of the pro‐inflammatory AP‐1 transcriptional targets IL‐6 and IL‐1β. RNA‐seq data suggested that the loss of Trem2 expression resulted in the pronounced upregulation of the IL‐6 family member Lif, which reportedly alleviates myocardial and renal fibrosis.^42^ Consistently, Lif stimulation for 48–72 h can reportedly suppress collagen expression and fibroblast transformation.^43^ In line with this prior evidence, in this study, Lif markedly suppressed dermal fibroblast proliferative activity, migration and phenotypic transformation. Both in vitro and in vivo experiments confirmed that Trem2 blocks the MAPK/AP‐1 axis to suppress Lif expression, thus suggesting that the loss of Trem2 can impair the healing of diabetic wounds at least in part due to the upregulation of Lif. An overview of the mechanism is shown in Figure 10. However, it also remains possible that other cytokines or signalling pathways may also underlie the role that Trem2 plays as a regulator of diabetic wound healing.

There are some limitations to this analysis. For one, while Trem2 can control the production of the secondary messengers IP_3_ and DAG,^44^ further work will be necessary to fully elucidate the nature of the crosstalk between Trem2 and the MAPK pathway. Moreover, while this study focused on the role that macrophage Trem2 plays as a regulator of Lif expression and associated skin fibroblast activity, these findings do not exclude the possibility that Trem2 may also regulate other cell populations including endothelial cells, keratinocytes and dendritic cells in the context of such wound healing. In recent studies, Maschalidi et al. have revealed the significant role of dendritic cells in the skin healing process of diabetes.^45^ To gain a comprehensive understanding of the pathogenesis of diabetic skin ulcers and explore novel therapeutic strategies, further investigation into the role of dendritic cell Trem2 in diabetic skin healing is imperative. Additional studies employing cell type‐ and disease stage‐specific Trem2 knockout models will be vital to more fully clarify the spatiotemporal dynamics and processes through which Trem2 shapes the healing of diabetic wounds. While the present results suggest that Trem2 upregulation is a relatively specific biomarker associated with wounding in diabetic model systems, this process is also likely to coincide with monocyte differentiation into macrophages and it thus remains to be determined as to whether Trem2 accumulates during this process of monocytic differentiation or their infiltration into the wound microenvironment. Accordingly, additional studies should be conducted with a focus on the transcriptional regulatory mechanisms and/or processes responsible for Trem2 protein stabilization in the context of tissue injury and wound healing.

In summary, this study provides novel evidence for the key role that the IL‐4‐Trem2‐MAPK‐AP‐1 axis plays in the regulation of macrophage functionality, suggesting that Trem2‐mediated crosstalk between myeloid cells and fibroblasts may be an essential facet of the diabetic wound healing process. These findings further suggest that in situ sTrem2 administration may represent an effective approach to treating non‐resolving wounds in patients with diabetes, while emphasizing the essential roles that macrophage Trem2 plays in the regulation of dermal fibroblast activity.

## AUTHOR CONTRIBUTIONS

*Xinlin Zhu, Chao Zhang and Weiwei Jiang*: contributed equally as co‐first authors to this study. *Xinlin Zhu, Chao Zhang, Weiwei Jiang, Wanqing Liao, Youming Chen, Wenjie Fang and Weihua Pan*: conceived and designed the research project. *Xinlin Zhu, Chao Zhang, Weiwei Jiang, Zhao Xiang zeng, Keming Zhang, Mingwei Du, Juan Chen, Qian Wu, and Youming Chen*: conducted the experiments and collected the data. *Xinlin Zhu and Zhaoxiang Zeng*: performed the data analysis. *Xinlin Zhu, Chao Zhang, Zhaoxiang Zeng, Youming Chen, Wenjie Fang and Weihua Pan*: contributed to the manuscript writing. All authors critically reviewed and approved the final version of the manuscript.

## CONFLICT OF INTEREST STATEMENT

The authors declare that they have no conflict of interest.

### ETHICS STATEMENT

All animal experiments conducted in this study were ethically reviewed and approved by the Animal Ethics Committee of Shanghai Changzheng Hospital, Naval Medical University. These experiments were carried out in strict accordance with the established ethical guidelines and principles for the care and use of laboratory animals. All efforts were made to ensure minimal suffering and the highest standards of animal welfare throughout the research process.

## Supporting information



Supplementary Material 1. Genotyping of TREM2‐cKO mice WT: one band with 278 bp; Heterozygous: two bands with 335 and 278 bp; Homozygous: one band with 335 bp.

Supplementary Material 2. The antibodies used in this study.

Supplementary Material 3. The primers used in this study are listed in Supplementary Material 3.

Supplementary Material 4. Analysis of trem2 expression in skin tissue of normal mice (A) and T2‐cKO mice (B) by flow cytometry.

Supplementary Material 5. (A) Images of histological staining and quantification of skin tissue. Scale bar is 100 µm. *n* = 5 samples/group. (B, C) Images of Masson staining and quantification of skin tissue. *n *= 5 samples/group.

Supplementary Material 6. Coomassie Blue Staining of GST and GST‐IL‐4 fusion protein.

Supplementary Material 7. (A) Volcano plot showing the expression patterns of differentially expressed genes post‐diabetic wound injury in T2‐flox and T2‐cKO mice. (B, C) AP‐1‐luciferase activity was measured by an AP‐1 transcription factor assay kit. *n* = 6 samples/group. *p < .05; **p < .01.

Supplementary Material 8. (A, B) Trem2 deficiency impaired the wound healing through enhancing Fosl1 and Junb expression. (C) Skin wound areas on the indicated days and quantification. *n* = 8 samples/group. (D, E) Images of histological staining on day 9 post‐injury. Scale bar is 100 µm. *n* = 8 samples/group. (F, G) Images of Masson staining and quantification on day 9 post‐injury. *n *= 8 samples/group. (H, I) Images of immunofluorescence staining of CD206, F4/80 and quantification on day 6 post‐injury. Scale bar is 100 µm. *n* = 6 samples/group. (J, K) Representative images and quantitative analysis showing the levels of superoxide anions as measured by DHE staining. *n* = 5 samples/group. (L, M) Images of immunofluorescence staining of α‐SMA and quantification on day 6 post‐injury. Scale bar is 100 µm. *n* = 6 samples/group. Images of immunofluorescence staining of CD31 and quantification on day 6 post‐injury. Scale bar is 100 µm. *n* = 6 samples/group.

Supplementary Material 9. (A, B) The expression of Lif was detected by Western blotting and the expression levels in the different groups were quantified. *n *= 5 samples/group.

## Data Availability

The data underlying this article will be shared on reasonable request to the corresponding authors.

## References

[1] Spampinato SF , Caruso GI , De Pasquale R , Sortino MA , Merlo S . The treatment of impaired wound healing in diabetes: looking among old drugs. Pharmaceuticals (Basel). 2020;13(4):60. 32244718 10.3390/ph13040060 PMC7243111

[2] Artasensi A , Pedretti A , Vistoli G , Fumagalli L . Type 2 diabetes mellitus: a review of multi‐target drugs. Molecules. 2020;25(8):1987. 32340373 10.3390/molecules25081987 PMC7221535

[3] Everett E , Mathioudakis N . Update on management of diabetic foot ulcers. Ann N Y Acad Sci. 2018;1411(1):153‐165. 29377202 10.1111/nyas.13569 PMC5793889

[4] Kavitha KV , Tiwari S , Purandare VB , Khedkar S , Bhosale SS , Unnikrishnan AG . Choice of wound care in diabetic foot ulcer: a practical approach. World J Diabetes. 2014;5(4):546‐556. 25126400 10.4239/wjd.v5.i4.546 PMC4127589

[5] Patel S , Srivastava S , Singh MR , Singh D . Mechanistic insight into diabetic wounds: pathogenesis, molecular targets and treatment strategies to pace wound healing. Biomed Pharmacother. 2019;112:108615. 30784919 10.1016/j.biopha.2019.108615

[6] Kim SY , Nair MG . Macrophages in wound healing: activation and plasticity. Immunol Cell Biol. 2019;97(3):258‐267. 30746824 10.1111/imcb.12236 PMC6426672

[7] Minutti CM , Knipper JA , Allen JE , Zaiss DM . Tissue‐specific contribution of macrophages to wound healing. Semin Cell Dev Biol. 2017;61:3‐11. 27521521 10.1016/j.semcdb.2016.08.006

[8] Krzyszczyk P , Schloss R , Palmer A , Berthiaume F . The role of macrophages in acute and chronic wound healing and interventions to promote pro‐wound healing phenotypes. Front Physiol. 2018;9:419. 29765329 10.3389/fphys.2018.00419 PMC5938667

[9] Buechler MB , Fu W , Turley SJ . Fibroblast‐macrophage reciprocal interactions in health, fibrosis, and cancer. Immunity. 2021;54(5):903‐915. 33979587 10.1016/j.immuni.2021.04.021

[10] Vannella KM , Wynn TA . Mechanisms of organ injury and repair by macrophages. Annu Rev Physiol. 2017;79:593‐617. 27959618 10.1146/annurev-physiol-022516-034356

[11] Li RY , Qin Q , Yang HC , et al. TREM2 in the pathogenesis of AD: a lipid metabolism regulator and potential metabolic therapeutic target. Mol Neurodegener. 2022;17(1):40. 35658903 10.1186/s13024-022-00542-y PMC9166437

[12] Ulland TK , Colonna M . TREM2 – a key player in microglial biology and Alzheimer disease. Nat Rev Neurol. 2018;14(11):667‐675. 30266932 10.1038/s41582-018-0072-1

[13] Painter MM , Atagi Y , Liu CC , et al. TREM2 in CNS homeostasis and neurodegenerative disease. Mol Neurodegener. 2015;10:43. 26337043 10.1186/s13024-015-0040-9 PMC4560063

[14] Yeh FL , Wang Y , Tom I , Gonzalez LC , Sheng M . TREM2 binds to apolipoproteins, including APOE and CLU/APOJ, and thereby facilitates uptake of amyloid‐beta by microglia. Neuron. 2016;91(2):328‐340. 27477018 10.1016/j.neuron.2016.06.015

[15] Park M , Yi JW , Kim EM , et al. Triggering receptor expressed on myeloid cells 2 (TREM2) promotes adipogenesis and diet‐induced obesity. Diabetes. 2015;64(1):117‐127. 25114293 10.2337/db13-1869

[16] Jaitin DA , Adlung L , Thaiss CA , et al. Lipid‐associated macrophages control metabolic homeostasis in a Trem2‐dependent manner. Cell. 2019;178(3):686‐698. e614. 31257031 10.1016/j.cell.2019.05.054 PMC7068689

[17] Wang X , He Q , Zhou C , et al. Prolonged hypernutrition impairs TREM2‐dependent efferocytosis to license chronic liver inflammation and NASH development. Immunity. 2023;56(1):58‐77. e11. 36521495 10.1016/j.immuni.2022.11.013 PMC9839616

[18] Seno H , Miyoshi H , Brown SL , Geske MJ , Colonna M , Stappenbeck TS . Efficient colonic mucosal wound repair requires Trem2 signaling. Proc Natl Acad Sci USA. 2009;106(1):256‐261. 19109436 10.1073/pnas.0803343106 PMC2629230

[19] Wiśniewski JR , Zougman A , Nagaraj N , Mann M . Universal sample preparation method for proteome analysis. Nat Methods. 2009;6(5):359‐362. 19377485 10.1038/nmeth.1322

[20] Sharifiaghdam M , Shaabani E , Faridi‐Majidi R , De Smedt SC , Braeckmans K , Fraire JC . Macrophages as a therapeutic target to promote diabetic wound healing. Mol Ther. 2022;30(9):2891‐2908. 35918892 10.1016/j.ymthe.2022.07.016 PMC9482022

[21] Dardenne C , Salon M , Authier H , et al. Topical aspirin administration improves cutaneous wound healing in diabetic mice through a phenotypic switch of wound macrophages toward an anti‐inflammatory and proresolutive profile characterized by LXA4 release. Diabetes. 2022;71(10):2181‐2196. 35796692 10.2337/db20-1245

[22] Kimball A , Schaller M , Joshi A , et al. Ly6C(Hi) blood monocyte/macrophage drive chronic inflammation and impair wound healing in diabetes mellitus. Arterioscler Thromb Vasc Biol. 2018;38(5):1102‐1114. 29496661 10.1161/ATVBAHA.118.310703 PMC5920725

[23] Sanyal S , Sandstrom DJ , Hoeffer CA , Ramaswami M . AP‐1 functions upstream of CREB to control synaptic plasticity in Drosophila. Nature. 2002;416(6883):870‐874. 11976688 10.1038/416870a

[24] Toone WM , Morgan BA , Jones N . Redox control of AP‐1‐like factors in yeast and beyond. Oncogene. 2001;20(19):2336‐2346. 11402331 10.1038/sj.onc.1204384

[25] Yu Y , Wang Y , Niu Y , Fu L , Chin YE , Yu C . Leukemia inhibitory factor attenuates renal fibrosis through Stat3‐miR‐29c. Am J Physiol Ren Physiol. 2015;309(7):F595‐F603. 10.1152/ajprenal.00634.2014 26155847

[26] Welc SS , Flores I , Wehling‐Henricks M , et al. Targeting a therapeutic LIF transgene to muscle via the immune system ameliorates muscular dystrophy. Nat Commun. 2019;10(1):2788. 31243277 10.1038/s41467-019-10614-1 PMC6594976

[27] Jung SH , Hwang BH , Shin S , et al. Spatiotemporal dynamics of macrophage heterogeneity and a potential function of Trem2(hi) macrophages in infarcted hearts. Nat Commun. 2022;13(1):4580. 35933399 10.1038/s41467-022-32284-2 PMC9357004

[28] Yunna C , Mengru H , Lei W , Weidong C . Macrophage M1/M2 polarization. Eur J Pharmacol. 2020;877:173090. 32234529 10.1016/j.ejphar.2020.173090

[29] Zhou X , Li W , Wang S , et al. YAP aggravates inflammatory bowel disease by regulating M1/M2 macrophage polarization and gut microbial homeostasis. Cell Rep. 2019;27(4):1176‐1189. e1175. 31018132 10.1016/j.celrep.2019.03.028

[30] Peng Q , Malhotra S , Torchia JA , Kerr WG , Coggeshall KM , Humphrey MB . TREM2‐ and DAP12‐dependent activation of PI3K requires DAP10 and is inhibited by SHIP1. Sci Signal. 2010;3(122):ra38. 20484116 10.1126/scisignal.2000500 PMC2900152

[31] Kober DL , Stuchell‐Brereton MD , Kluender CE , et al. Functional insights from biophysical study of TREM2 interactions with apoE and Aβ(1‐42). Alzheimers Dement. 2020;3:1‐14. 10.1002/alz.12194 PMC8026773 33090700

[32] Xie X , Zhang W , Xiao M , et al. TREM2 acts as a receptor for IL‐34 to suppress acute myeloid leukemia in mice. Blood. 2023;141(26):3184‐3198. 37001042 10.1182/blood.2022018619 PMC10646818

[33] Zenz R , Eferl R , Scheinecker C , et al. Activator protein 1 (Fos/Jun) functions in inflammatory bone and skin disease. Arthritis Res Ther. 2008;10(1):201. 18226189 10.1186/ar2338 PMC2374460

[34] Schonthaler HB , Guinea‐Viniegra J , Wagner EF . Targeting inflammation by modulating the Jun/AP‐1 pathway. Ann Rheum Dis. 2011;70:i109‐112. Suppl 1. 21339212 10.1136/ard.2010.140533

[35] Matthews CP , Colburn NH , Young MR . AP‐1 a target for cancer prevention. Curr Cancer Drug Targets. 2007;7(4):317‐324. 17979626 10.2174/156800907780809723

[36] Baumann D , Drebant J , Hagele T , et al. p38 MAPK signaling in M1 macrophages results in selective elimination of M2 macrophages by MEK inhibition. J Immunother Cancer. 2021;9(7):e002319. 34285105 10.1136/jitc-2020-002319 PMC8292803

[37] Neamatallah T . Mitogen‐activated protein kinase pathway: a critical regulator in tumor‐associated macrophage polarization. J Microsc Ultrastruct. 2019;7(2):53‐56. 31293885 10.4103/JMAU.JMAU_68_18 PMC6585481

[38] Ren M , Guo Y , Wei X , et al. TREM2 overexpression attenuates neuroinflammation and protects dopaminergic neurons in experimental models of Parkinson's disease. Exp Neurol. 2018;302:205‐213. 29407460 10.1016/j.expneurol.2018.01.016

[39] Zhang J , Liu Y , Zheng Y , et al. TREM‐2‐p38 MAPK signaling regulates neuroinflammation during chronic cerebral hypoperfusion combined with diabetes mellitus. J Neuroinflam. 2020;17(1):2. 10.1186/s12974-019-1688-9 PMC6942413 31900229

[40] Brazil JC , Quiros M , Nusrat A , Parkos CA . Innate immune cell‐epithelial crosstalk during wound repair. J Clin Invest. 2019;129(8):2983‐2993. 31329162 10.1172/JCI124618 PMC6668695

[41] Burgess JL , Wyant WA , Abdo Abujamra B , Kirsner RS , Jozic I . Diabetic wound‐healing science. Medicina (Kaunas). 2021;57(10):1072. 34684109 10.3390/medicina57101072 PMC8539411

[42] Zou Y , Takano H , Mizukami M , et al. Leukemia inhibitory factor enhances survival of cardiomyocytes and induces regeneration of myocardium after myocardial infarction. Circulation. 2003;108(6):748‐753. 12860906 10.1161/01.CIR.0000081773.76337.44

[43] Wang F , Trial J , Diwan A , et al. Regulation of cardiac fibroblast cellular function by leukemia inhibitory factor. J Mol Cell Cardiol. 2002;34(10):1309‐1316. 12392991 10.1006/jmcc.2002.2059

[44] Thatcher JD . The inositol trisphosphate (IP3) signal transduction pathway. Sci Signal. 2010;3(119):tr3. 20424267 10.1126/scisignal.3119tr3

[45] Maschalidi S , Mehrotra P , Keçeli BN , et al. Targeting SLC7A11 improves efferocytosis by dendritic cells and wound healing in diabetes. Nature. 2022;606(7915):776‐784. 35614212 10.1038/s41586-022-04754-6

