# Supplementary figures and images for: Trem2 acts as a non‐classical receptor of interleukin‐4 to promote diabetic wound healing
Source: Clin Transl Med. 2024 Sep 30;14(10):e70026. doi: 10.1002/ctm2.70026 (PMC11442487; doi:10.1002/ctm2.70026)

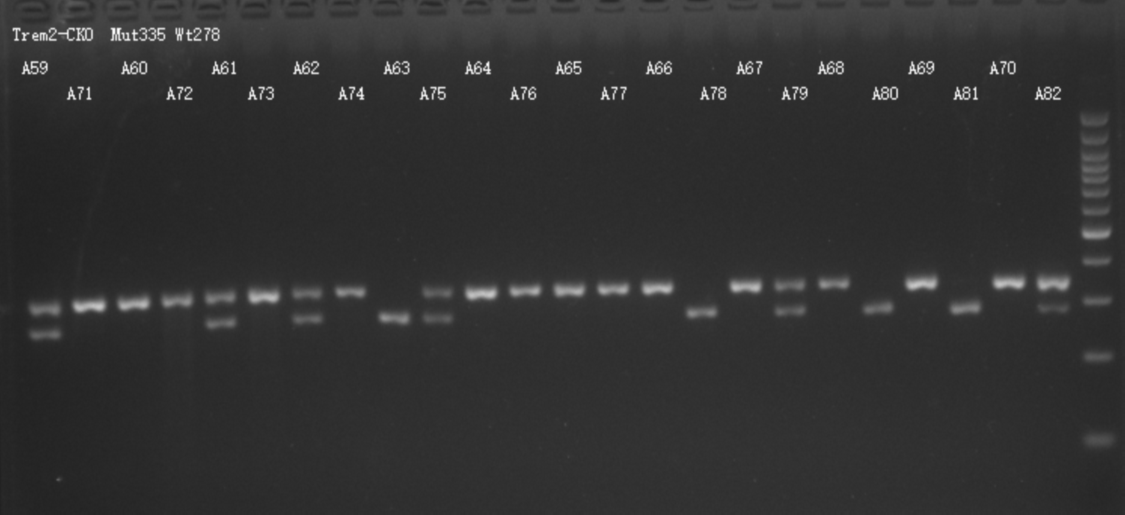

Supplement: Supplementary file 1 — Supplementary Material 1. Genotyping of TREM2‐cKO mice WT: one band with 278 bp; Heterozygous: two bands with 335 and 278 bp; Homozygous: one band with 335 bp. [file CTM2-14-e70026-s007.png]

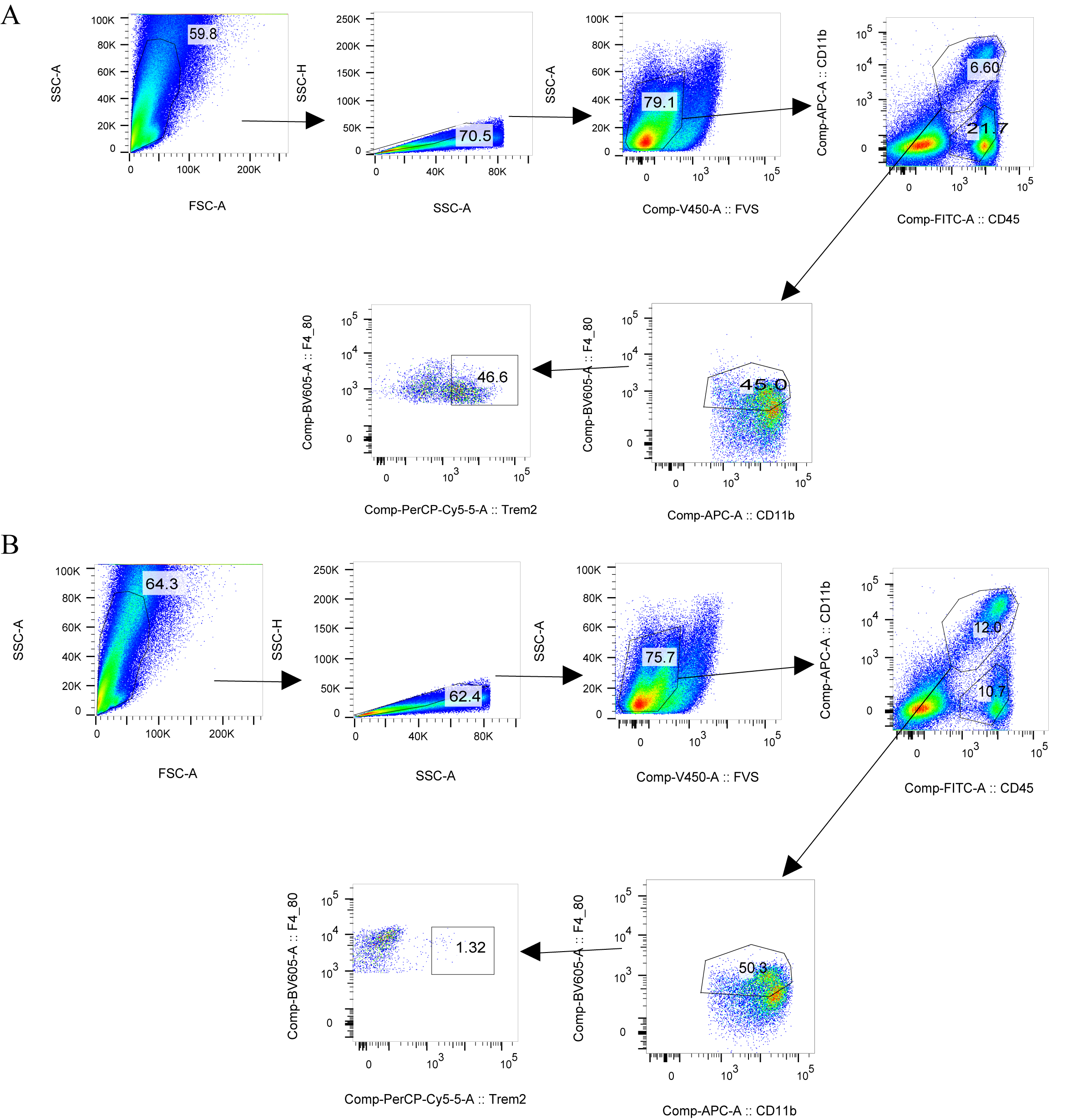

Supplement: Supplementary file 4 — Supplementary Material 4. Analysis of trem2 expression in skin tissue of normal mice (A) and T2‐cKO mice (B) by flow cytometry. [file CTM2-14-e70026-s008.tif]

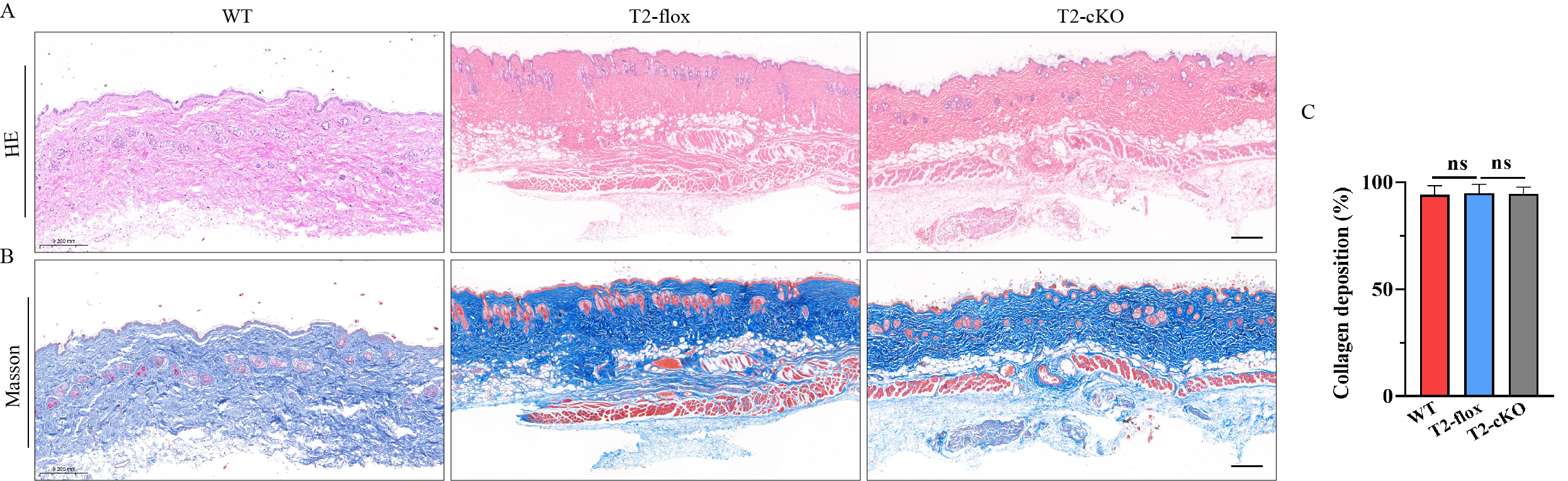

Supplement: Supplementary file 5 — Supplementary Material 5. (A) Images of histological staining and quantification of skin tissue. Scale bar is 100 µm. n = 5 samples/group. (B, C) Images of Masson staining and quantification of skin tissue. n = 5 samples/group. [file CTM2-14-e70026-s001.jpg]

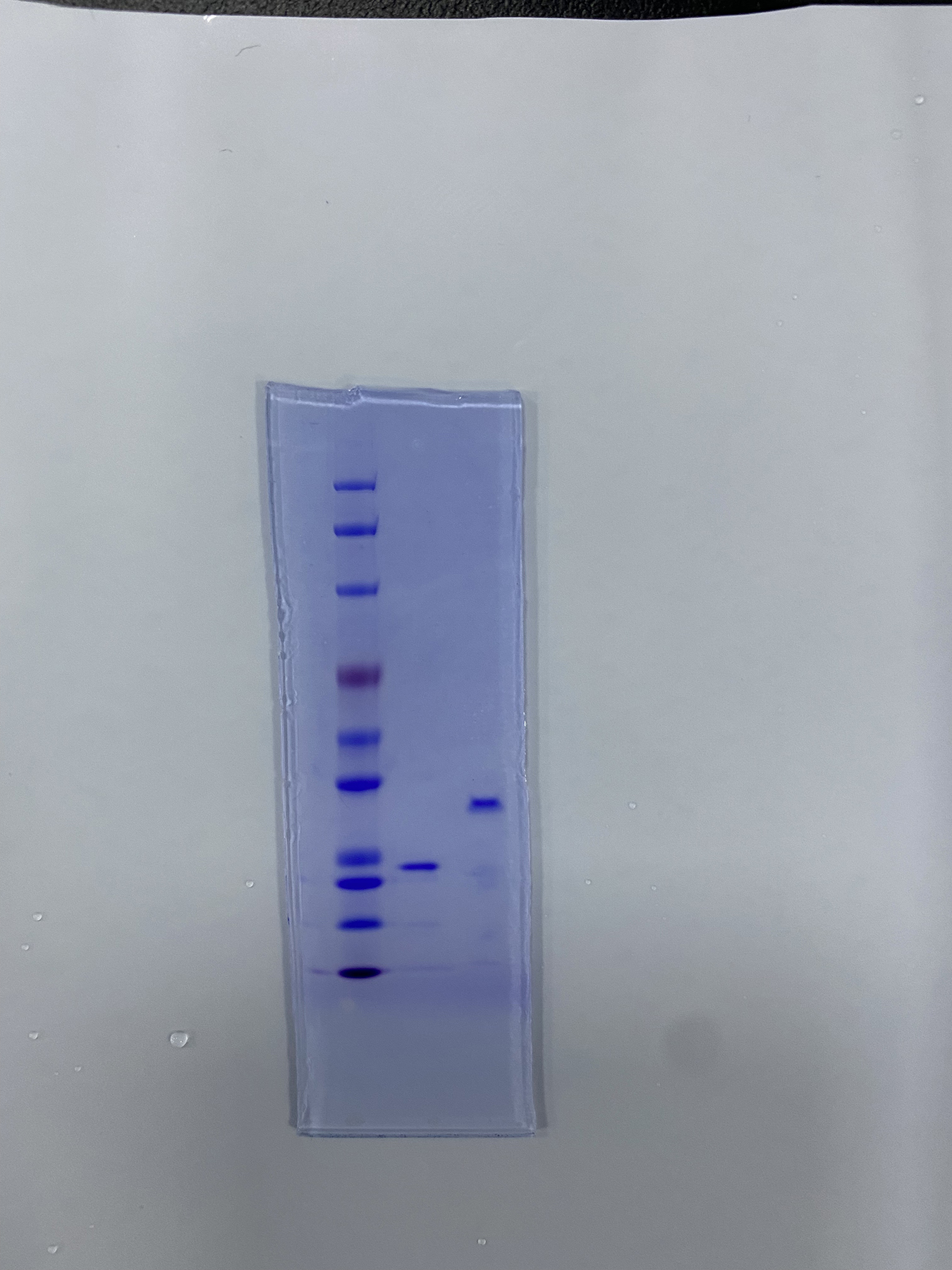

Supplement: Supplementary file 6 — Supplementary Material 6. Coomassie Blue Staining of GST and GST‐IL‐4 fusion protein. [file CTM2-14-e70026-s005.jpg]

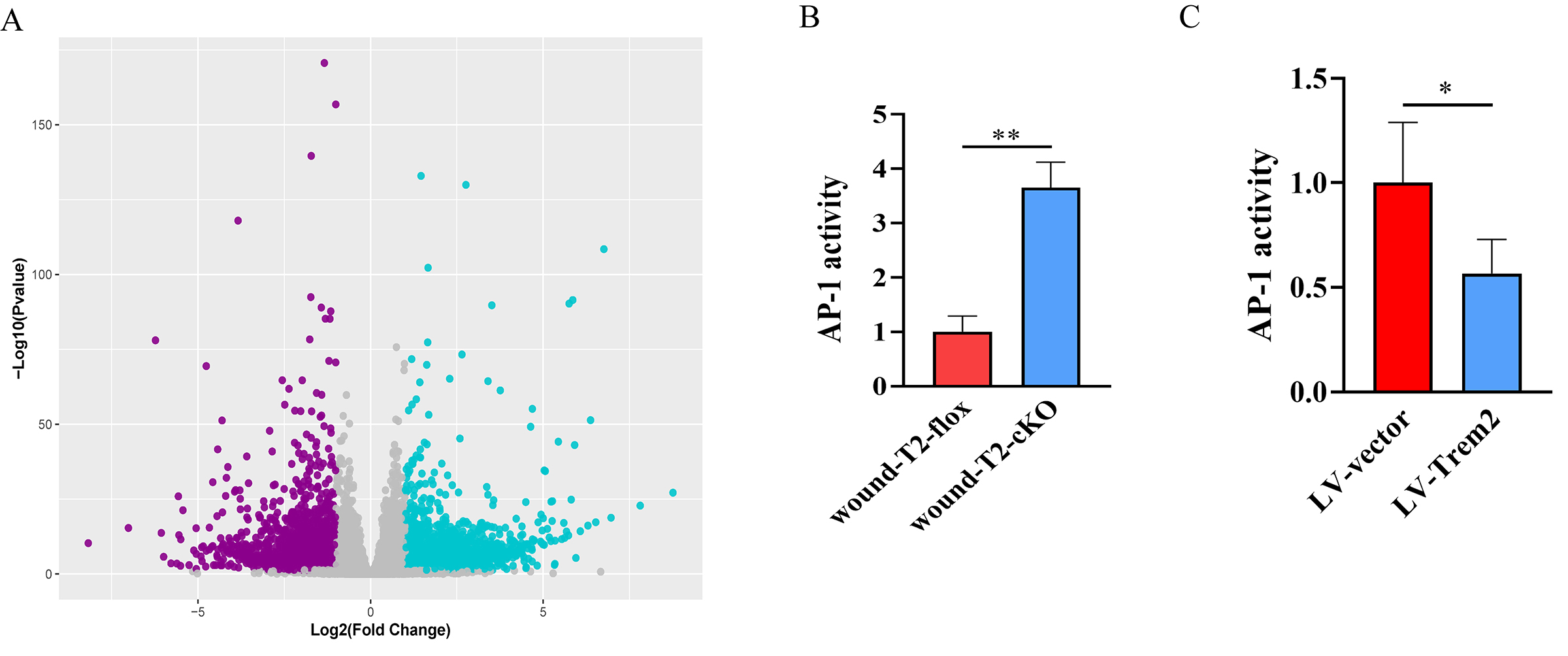

Supplement: Supplementary file 7 — Supplementary Material 7. (A) Volcano plot showing the expression patterns of differentially expressed genes post‐diabetic wound injury in T2‐flox and T2‐cKO mice. (B, C) AP‐1‐luciferase activity was measured by an AP‐1 transcription factor assay kit. n = 6 samples/group. *p < .05; **p < .01. [file CTM2-14-e70026-s009.jpg]

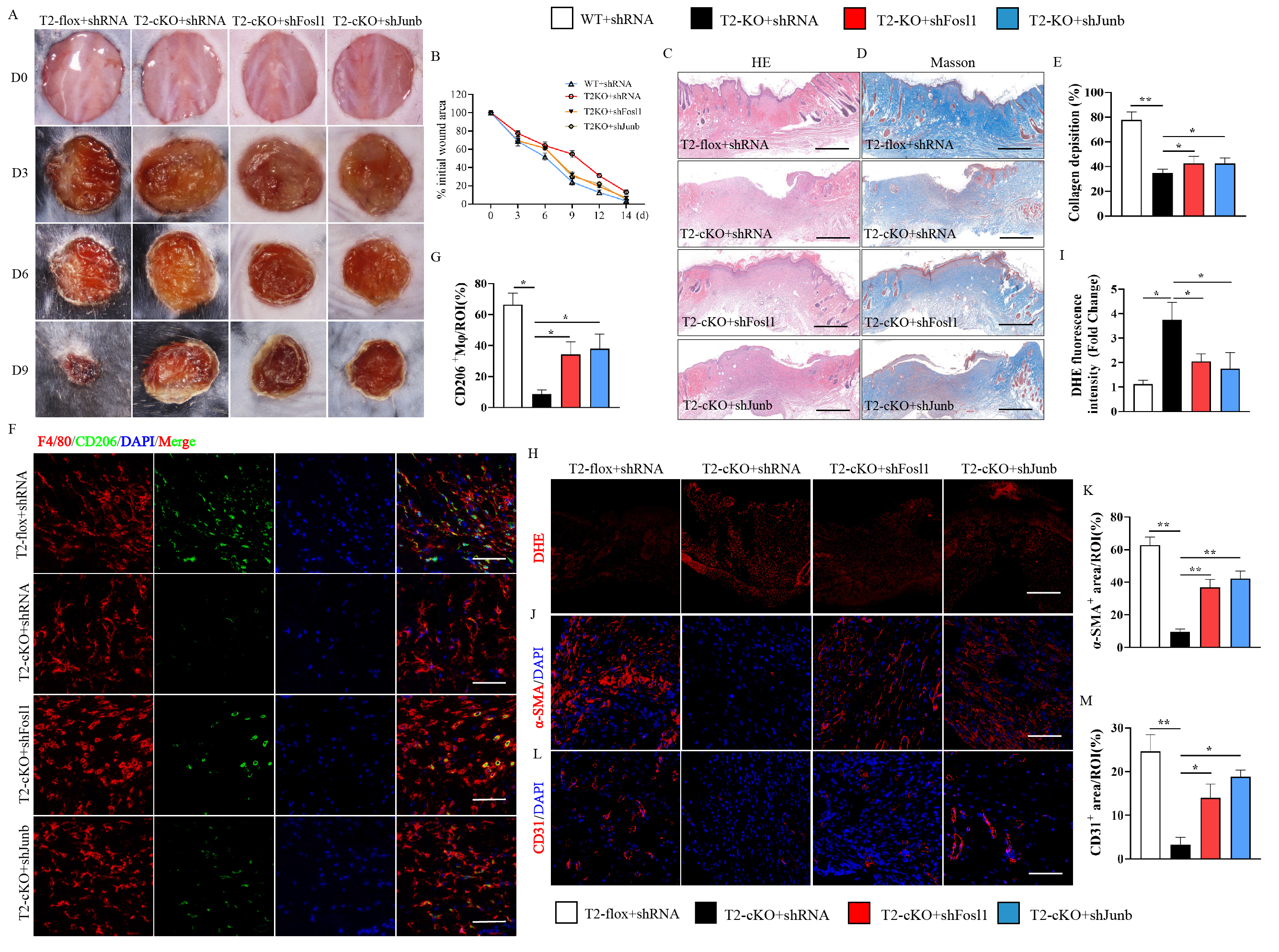

Supplement: Supplementary file 8 — Supplementary Material 8. (A, B) Trem2 deficiency impaired the wound healing through enhancing Fosl1 and Junb expression. (C) Skin wound areas on the indicated days and quantification. n = 8 samples/group. (D, E) Images of histological staining on day 9 post‐injury. Scale bar is 100 µm. n = 8 samples/group. (F, G) Images of Masson staining and quantification on day 9 post‐injury. n = 8 samples/group. (H, I) Images of immunofluorescence staining of CD206, F4/80 and quantification on day 6 post‐injury. Scale bar is 100 µm. n = 6 samples/group. (J, K) Representative images and quantitative analysis showing the levels of superoxide anions as measured by DHE staining. n = 5 samples/group. (L, M) Images of immunofluorescence staining of α‐SMA and quantification on day 6 post‐injury. Scale bar is 100 µm. n = 6 samples/group. Images of immunofluorescence staining of CD31 and quantification on day 6 post‐injury. Scale bar is 100 µm. n = 6 samples/group. [file CTM2-14-e70026-s004.jpg]

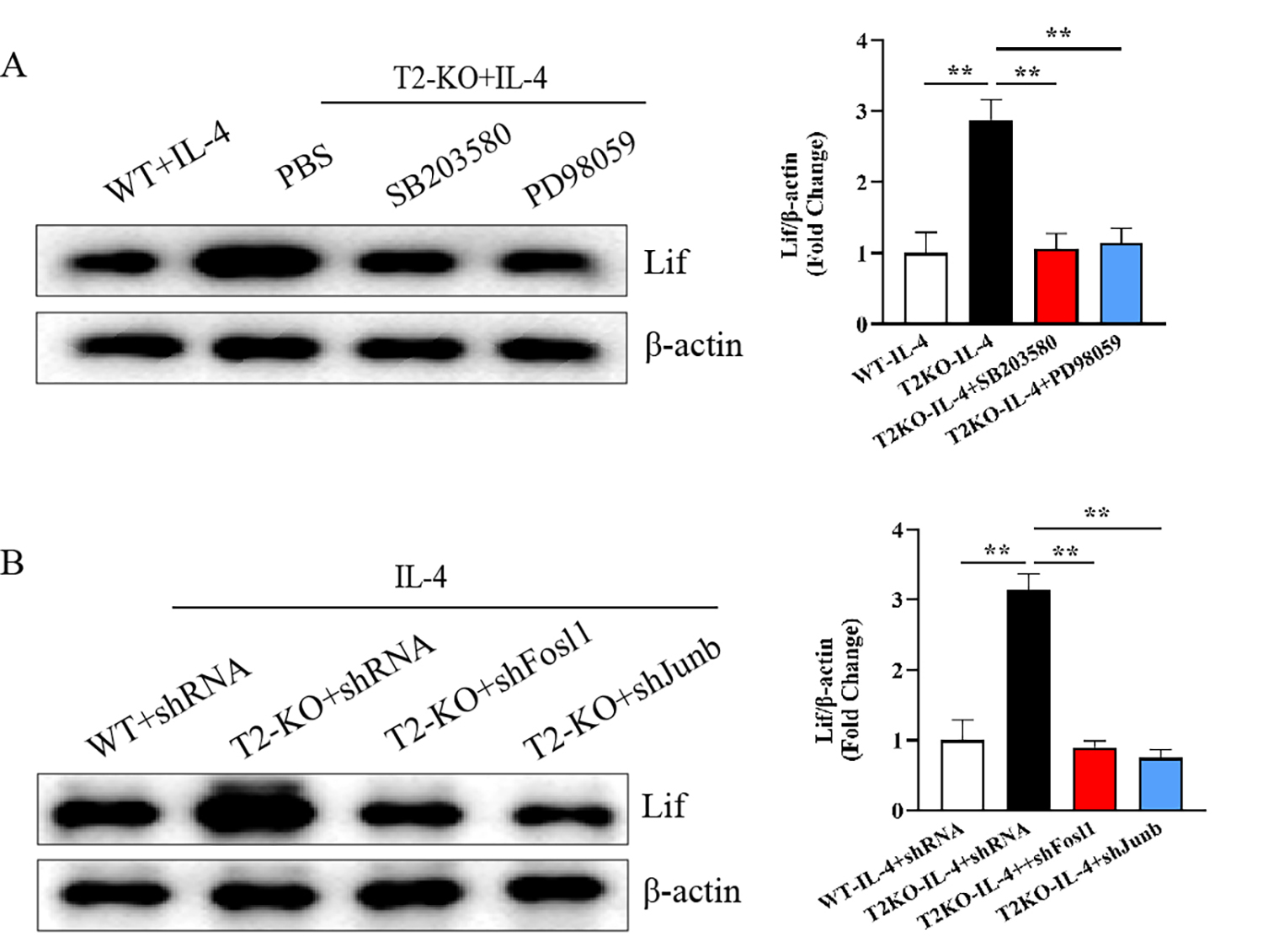

Supplement: Supplementary file 9 — Supplementary Material 9. (A, B) The expression of Lif was detected by Western blotting and the expression levels in the different groups were quantified. n = 5 samples/group. [file CTM2-14-e70026-s006.jpg]
