# Supplementary material for: Trem2 acts as a non‐classical receptor of interleukin‐4 to promote diabetic wound healing
Source: Clin Transl Med. 2024 Sep 30;14(10):e70026. doi: 10.1002/ctm2.70026 (PMC11442487; doi:10.1002/ctm2.70026)
Supplement: Supplementary file 2 — Supplementary Material 2. The antibodies used in this study. [file CTM2-14-e70026-s003.docx]

**Supplemental Table 2A .** The antibodies used in IF and IHC analysis.

| **Brand** | **Name** | **Catalog** | **Dilution ratio** |
| --- | --- | --- | --- |
| CST | Trem2 | 91068 | 1:500 |
| ABclonal | GPX4 | A21440 | 1:50 |
| ABclonal | IL-6 | A11114 | 1:50 |
| Proteintech | IL-4 | 66142-1-lg | 1:200 |
| Abcam | IL-1β | ab283818 | 1:500 |
| Abcam | TNF-α | ab183218 | 1:2000 |
| Abcam | α-SMA | ab7817 | 1:1000 |
| Abcam | CD31 | ab222783 | 1:100 |
| Abcam | F4/80 | ab6640 | 1:200 |
| Abcam | CD206 | ab64693 | 1:1000 |
| Abcam | Arg1 | ab239731 | 1:200 |
| Abcam | CD86 | ab119857 | 1:200 |
| Servicebio | Cy3 conjugated Goat Anti-mouse IgG | GB21301 | 1:300 |
| Servicebio | Cy3-conjugated AffiniPure Goat Anti-Rat IgG | GB21302 | 1:300 |
| Servicebio | FITC conjugated Donkey Anti-Rabbit IgG | GB22403 | 1:200 |
| Servicebio | HRP conjugated Goat Anti-Rabbit IgG | GB23303 | 1:400 |
| Servicebio | Cy3 conjugated Goat Anti-Rabbit IgG | GB21303 | 1:300 |
| Servicebio | FITC conjugated Goat Anti-Rat IgG | GB22302 | 1:200 |
| Servicebio | Alexa Fluor® 488-conjugated Goat Anti-Rabbit IgG | GB25303 | 1:400 |
| Servicebio | FITC conjugated Donkey Anti-Mouse IgG | GB22401 | 1:200 |

**Supplemental Table 2B .** The primary antibodies used in Western blot analysis.

| **Brand** | **Name** | **Catalog** | **Dilution ratio** |
| --- | --- | --- | --- |
| Abcam | collagen I | ab138492 | 1:1000 |
| Abcam | collagen Ⅲ | ab184993 | 1:1000 |
| Abcam | α-SMA | ab7817 | 1:1000 |
| Abcam | Nox2 | ab310337 | 1:1000 |
| Abcam | IL-6 | ab290735 | 1:1000 |
| Abcam | Txnip | ab210826 | 1:200 |
| Abcam | IL-1β | ab283818 | 1:1000 |
| Abcam | TGF-β1 | ab215715 | 1:1000 |
| Abcam | vimentin | ab92547 | 1:2000 |
| Abcam | β-actin | ab8226 | 1:1000 |
| CST | Trem2 | 91068 | 1:1000 |
| CST | p-Fos1 | 5348 | 1:1000 |
| CST | Fos1 | 31254 | 1:1000 |
| CST | p-Junb | 8053 | 1:1000 |
| CST | Junb | 3753 | 1:1000 |
| CST | p-p38 | 9211 | 1:1000 |
| CST | p38 | 9212 | 1:1000 |
| CST | p-ERK1/2 | 4377 | 1:1000 |
| CST | ERK1/2 | 9102 | 1:1000 |
| CST | p-JNK1/2 | 9251 | 1:1000 |
| CST | JNK1/2 | 67096 | 1:1000 |

CST：Cell Signaling Technology
