# Supplementary material for: Trem2 acts as a non‐classical receptor of interleukin‐4 to promote diabetic wound healing
Source: Clin Transl Med. 2024 Sep 30;14(10):e70026. doi: 10.1002/ctm2.70026 (PMC11442487; doi:10.1002/ctm2.70026)
Supplement: Supplementary file 3 — Supplementary Material 3. The primers used in this study are listed in Supplementary Material 3. [file CTM2-14-e70026-s002.docx]

| Gene | Forward Primer | Reverse Primer |
| --- | --- | --- |
| Trem2 | CTGGAACCGTCACCATCACTC | CGAAACTCGATGACTCCTCGG |
| Fosl1 | ATGTACCGAGACTACGGGGAA | CTGCTGCTGTCGATGCTTG |
| Junb | TCACGACGACTCTTACGCAG | CCTTGAGACCCCGATAGGGA |
| Fosl2 | CACGCCGAGTCCTACTCCA | GTGGGCTGTACCATCCACTG |
| Fos | CGGGTTTCAACGCCGACTA | TGGCACTAGAGACGGACAGAT |
| Fosb | CCTCCGCCGAGTCTCAGTA | CCTGGCATGTCATAAGGGTCA |
| Jund | GAAACGCCCTTCTATGGCGA | CAGCGCGTCTTTCTTCAGC |
| Jun | TTCCTCCAGTCCGAGAGCG | TGAGAAGGTCCGAGTTCTTGG |

Supplementary material 3
